# Proteomic analysis of exported chaperone/co-chaperone complexes of *P. falciparum* reveals an array of complex protein-protein interactions

**DOI:** 10.1038/srep42188

**Published:** 2017-02-20

**Authors:** Qi Zhang, Cheng Ma, Alexander Oberli, Astrid Zinz, Sonja Engels, Jude M. Przyborski

**Affiliations:** 1 Department of Parasitology, Philipps University Marburg, Marburg, Germany; 2 Center for Diagnostics & Therapeutics, Department of Chemistry, Georgia State University, Atlanta, Georgia, USA; 3 Swiss TPH Socinstrasse 57, CH 4002, Basel, Switzerland; 4 University of Basel, Petersplatz 1, CH 4001 Basel, Switzerland

## Abstract

Malaria parasites modify their human host cell, the mature erythrocyte. This modification is mediated by a large number of parasite proteins that are exported to the host cell, and is also the underlying cause for the pathology caused by malaria infection. Amongst these proteins are many Hsp40 co-chaperones, and a single Hsp70. These proteins have been implicated in several processes in the host cell, including a potential role in protein transport, however the further molecular players in this process remain obscure. To address this, we have utilized chemical cross-linking followed by mass spectrometry and immunoblotting to isolate and characterize proteins complexes containing an exported Hsp40 (PFE55), and the only known exported Hsp70 (PfHsp70x). Our data reveal that both of these proteins are contained in high molecular weight protein complexes. These complexes are found both in the infected erythrocyte, and within the parasite-derived compartment referred to as the parasitophorous vacuole. Surprisingly, our data also reveal an association of PfHsp70x with components of PTEX, a putative protein translocon within the membrane of the parasitophorous vacuole. Our results suggest that the *P. falciparum*- infected human erythrocyte contains numerous high molecular weight protein complexes, which may potentially be involved in host cell modification.

Despite concerted world-wide efforts, malaria remains one of the most deadly infectious diseases in the world, responsible for over 200 million infections and 430 000 human deaths annually, 70% of which are in children under 5 years of age and most of which occur in sub-Saharan Africa^1^. Pathology in the human host is caused by parasite stages that replicate within mature human red blood cells. Red blood cells themselves are less than ideal as a host cell, as they are metabolically quiescent. To survive and propagate within this barren host cell, the parasite is forced to take the initiative, and changes the structural and biochemical properties of the host cell to ensure its own survival^2^. For example, red blood cells infected with *P. falciparum* acquire the ability to adhere to endothelial cells in the peripheral circulation, thus avoiding passage through, and potential elimination in the spleen. This phenomenon, referred to as cytoadherance, is responsible for much of the pathology associated with malaria infection as it disrupts the microcirculation and leads to hypoxia in tissues and organs^3^. Over the past decades, we have gained a better understanding of how the parasite modifies the host cell. Of importance in this process seems to be a large number (>400) of proteins that the parasite synthesizes and transports to the host cell cytosol^2^,^4^,^5^,^6^. To reach the host cell, these proteins must pass through the parasitophorous vacuole (PV), a membrane-bound compartment generated by the parasite during the invasion process^7^. Once within the host cell, these proteins appear to mediate various aspects of host cell remodeling, including modulation of host cell stiffness, alterations in permeability of the host cell membrane to various solutes and cytoadherance^2^. Also involved in these processes are novel parasite-induced structures which appear in the infected cell, including Maurer’s clefts, the tubulovesicular membrane network, and J- and K- dots^7^,^8^,^9^. J-dots are highly mobile structures within the host cell cytosol, and have previously been shown to contain both the exported parasite chaperone PfHsp70x, and several exported Hsp40 co-chaperones, PFE55 and PFA660^8^,^10^. J-dots have also been shown to contain the exported parasite antigen PfEMP1, which has been implicated in cytoadherance of infected erythrocytes and thus pathology^8^. Whereas the co-chaperones PFE55 and PFA660 appear to be restricted to J-dots, PfHsp70x is dually localized to both the J-dots and the lumen of the parasitophorous vacuole, suggesting distinct functions at different locations^8^,^10^. One possibility is that PfHsp70x is involved in keeping exported proteins in a “translocation competent state” for passage through the vacuolar translocon PTEX^11^,^12^. Therefore, both PfHsp70x and exported Hsp40s have been proposed to be involved in protein traffic through the infected red blood cell, but additional molecular players involved in these processes have not been identified, and thus potential functional interactions have yet to be revealed. To address this, here we have used various proteomic methods to identify and characterize the molecular composition of J-dots, and identify several new J-dot proteins. Additionally, by analysis of crosslinked and immunoprecipitated protein complexes, we reveal the presence of at least three high molecular weight complexes containing PfHsp70x but of differing further molecular composition. These complexes are found within the parasitophorous vacuole and J-dots. Our data suggests that protein networks both within the infected erythrocyte cytosol, and the parasitophorous vacuole, are highly complex and potentially involved in multiple processes that may be essential for parasite survival.

## Results

### Hypotonic lysis followed by differential ultra-centrifugation identifies a fraction enriched in PfHsp70x/PfHsp40 complexes

**Figure 1:**
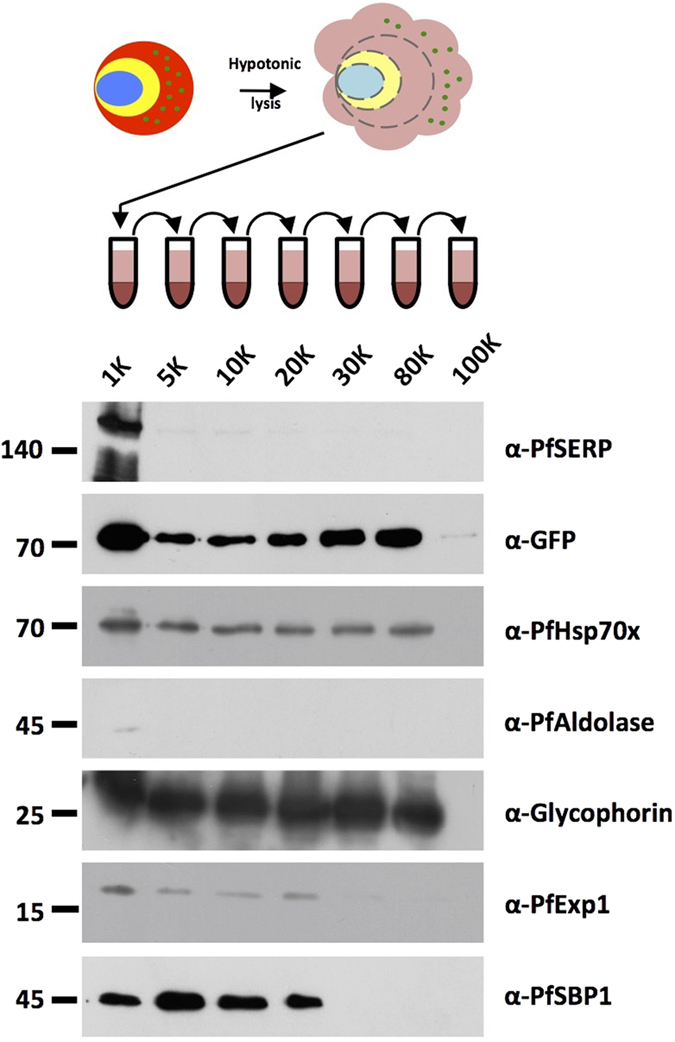
Differential centrifugation isolates an enriched J-dot fraction. Size markers in kDa. Antibodies used for immunodetection are indicated on right. Full-length blots are presented in the Supplementary Information file.

In a previous study we could demonstrate that J-dots are retained within the pellet fraction following lysis of the erythrocyte plasma membrane with the bacterial pore forming protein streptolysin O (SLO), suggesting that they are too large to pass through the *circa* 30 nm pores generated by this reagent. However, the same study could show that J-dots are released into the extracellular medium upon cell lysis by other methods^8^. For this reason, we decided to employ a two-step lysis/centrifugation protocol to isolate a J-dot enriched fraction for proteomic analysis. Erythrocytes infected with early trophozoite stage parasites expressing a GFP-tagged version of the J-dot co-chaperone PFE55 (PFE55^GFP^) were lysed hypotonically, and subjected to sequential differential centrifugation (Fig. 1). Equal cell equivalents of the post-centrifugation pellet were then subjected to Immonoblot analysis using antibodies raised against markers for various cellular membranes and compartments. PFE55^GFP^ and PfHsp70x could be detected in all pellet fractions from 1000–80000 *g*, but appear enriched in the 80000 *g* pellet (Fig. 1). Although a strong signal was also seen for these proteins in the 1000 *g* pellet, we suspect that this fraction contains a number of non-lysed cells, a hypothesis confirmed by the presence of both PfSERP (a soluble PV protein) and small amounts of Pfaldolase (a soluble protein of the parasite cytosol). The 80000 *g* pellet was devoid of PfSBP1 (a membrane bound marker of the Maurer’s clefts) and PfExp1 (a membrane bound marker of the parasitophorous vacuolar membrane, PVM), suggesting that it did not contain substantial amounts of material derived from these membranes. However, we were able to detect human glycophorin, showing that this fraction also contained some fragments of erythrocyte plasma membrane.

### The 80000 g fraction contains several high molecular weight complexes containing PFE55^GFP^ and PfHsp70x

**Figure 2:**
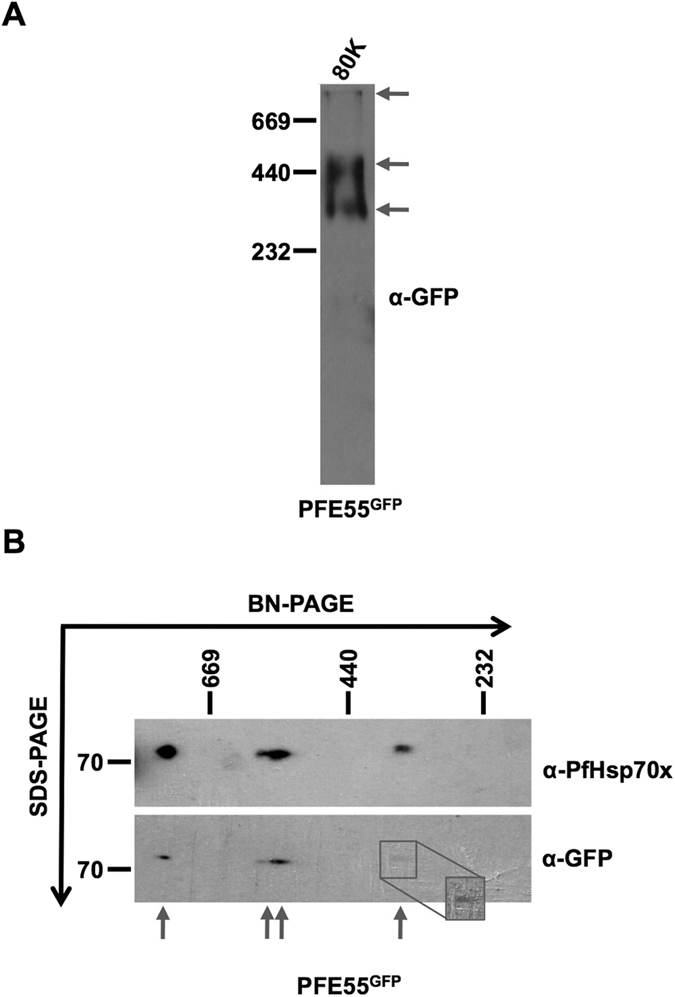
2D blue native/SDS-PAGE of an enriched J-dot fraction identifies high molecular weight complex containing PFE55^GFP^ and PfHsp70x. (**A**) Immunodetection of PFE55^GFP^ following blue native separation. Three bands can be visualized, referring to distinct high molecular weight complexes (indicated by arrows). (**B**) Second dimension SDS separation followed by immunodetection demonstrates the presence of PfHsp70x and PFE55^GFP^ in four distinct high molecular weight complexes (indicated by arrows). Inset shows higher contrast view. In (**A**,**B**) size markers in kDa. Antibodies used for immunodetection are indicated on right. Full-length blots are presented in the Supplementary Information file.

As our aim was to identify protein complexes containing chaperones/co-chaperones, we then carried out blue-native PAGE analysis of the 80000 *g* pellet fraction, followed by immonoblotting to identify complexes containing PFE55^GFP^. This analysis revealed that PFE55^GFP^ could be detected in two significant complexes (found at >440 kDa and >232 kDa, Fig. 2A). Additionally, a possible third high molecular weight complex could be detected at >669 kDa. A previous study from our group using co-IP analysis identified PfHsp70x as an interaction partner of PFE55^GFP^. To investigate if these two proteins are found together in high molecular weight complexes, we carried out a 2D Blue native/SDS-PAGE analysis of the 80000 *g* fraction. In the first dimension, native complexes are separated by their molecular mass. In a second denaturing step, individual proteins are then separated according to their molecular mass. We then detected PFE55^GFP^ and PfHsp70x using specific antibodies. We could detect PFE55^GFP^ in three complexes, running at a similar molecular mass as in the initial BN-gel (>669, >440 and a less abundant complex at >232 kDa (see high contrast insert, Fig. 2B). Significantly, PfHsp70x could also be found in complexes of similar molecular masses (Fig. 2B). These results suggest that PFE55^GFP^ and PfHsp70x are found in common high molecular weight complexes.

### Crosslinking followed by mass spectrometry identifies novel components of PFE55- and PfHsp70x- containing complexes

Our data strongly suggested that both PFE55^GFP^ and PfHsp70x are found in high molecular weight complexes within the infected host cell. To elucidate the composition of these complexes, we carried out chemical crosslinking followed by LC-MS/MS to find interaction partners of both PFE55^GFP^ and PfHsp70x. Erythrocytes infected with early trophozoite stage parasites expressing PFE55^GFP^ were crosslinked with 1 mM DSP, lysed and then protein complexes were isolated by immunoprecipitation (IP) using either anti-GFP or anti-PfHsp70x antibodies. Wild-type 3D7 strain parasites and bead-only immunoprecipitations were used as negative controls. The resulting immunoprecipitate was separated by SDS-PAGE, subjected to in-gel digestion with trypsin and identified by LC-MS/MS. All the protein identified were using ≥2 unique peptides matching, in total 41 *P. falciparum* proteins were identified by pulling down with anti-GFP, and 162 *P. falciparum* proteins were identified by pulling down with anti-PfHsp70x (Supplementary Table S1). To allow us to concentrate on potential interactions of high confidence, we then analysed the proteins for the presence of signals that would suggest either a secreted (i.e PV localization) or exported localization (i.e within the infected erythrocyte). Thus sequences were screened for the presence of (i) a classical N-terminal signal sequence only, (ii) a predicted PEXEL/HT export motif OR an annotation as “exported”, (iii) a hydrophobic domain. For PFE55^GFP^, 4 potential interactors contain a PEXEL/HT motif, 3 contain only an N-terminal signal sequence, and 2 a hydrophobic domain. 31 proteins do not contain any of these predicted trafficking or localization signals. For PfHsp70x, 8 potential interactors are predicted to possess a PEXEL/HT motif, 20 only an N-terminal signal sequence, and 8 a hydrophobic domain. The remaining 125 proteins do not contain any of these predicted trafficking or localization signals. In both cases, many of the proteins lacking any trafficking signals belonged to the parasites protein translation machinery that are often found as contaminants in proteomic analyses due to their high abundance, and for this reason we excluded these proteins from further analysis. To prioritize further identified proteins for analysis, we excluded any proteins with a ratio of (peptides IP/peptides control) <2. We then carried out literature searches to identify proteins with previously characterized localizations (Table 1). This left us with 41 proteins for further analysis (Table 1). We were particularly interested in further investigating proteins for which no previous localization has been determined.

Our previous published results, and those from both differential centrifugation and native PAGE, strongly suggested that PfHsp70x and PFE55^GFP^ are part of a common complex. In support of this hypothesis and as verification of our experimental strategy, mass spectrometry identified PFE55^GFP^ (albeit with a low number of peptides) as a crosslink product of PfHsp70x, and *vice versa*.

In addition to parasite encoded-proteins, we also identified a large number of human proteins in our analyses (Supplementary Table S2). Of greatest interest was the identification of an HsHsp70 homologue (although with a low number of peptides) as a potential interaction partner of the exported Hsp40 co-chaperone PFE55^GFP^ (Supplementary Table S2).

### Verification of complexes containing PfHsp70x

To verify potential interactions identified by mass spectrometry, we carried out chemical cross-linking as before, followed by immunoprecipitation using anti-PfHsp70x specific antisera and immunoblotting using specific antisera. As a control for non-specific interactions, we carried out the immunoprecipitation protocol, but without adding antibody. Please note that this analysis, and all further analyses, do not allow us to identify direct protein-protein interactions, but merely characterise the proteins composition of complexes containing the protein of interest. Additionally, our analyses do not distinguish between the isolation of a single, or multiple protein complexes.

**Figure 3:**
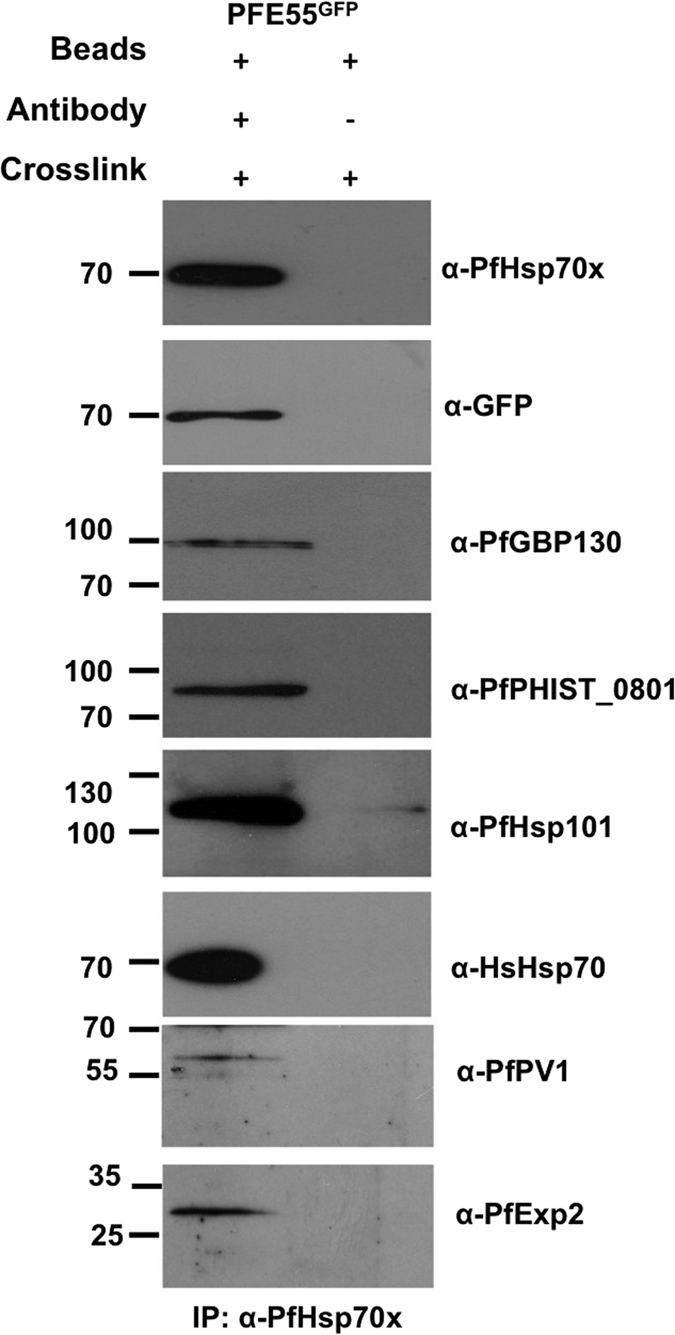
Immunodetection of anti-PfHsp70x co-IP verifies mass spectrometry data. Size markers in kDa. Antibodies used for immunodetection are indicated on right. Full-length blots are presented in the Supplementary Information file.

This analysis reveals that PfHsp70x is incorporated into a complex or complexes containing PFE55^GFP^, PfGBP130, PfPHIST_0801, PfHsp101, HsHsp70, PfPV1, and PfExp2 (Fig. 3).

### Verification of complexes containing PFE55^GFP^

As above, we wished to verify our mass spectrometry results by a secondary method. To this end we carried out chemical cross-linking as before, followed by immunoprecipitation using anti-GFP antibodies and Immonoblotting using specific antisera. As a control for non-specific interactions, we carried out the immunoprecipitation protocol on crosslinked samples prepared from wild-type 3D7 parasites.

**Figure 4:**
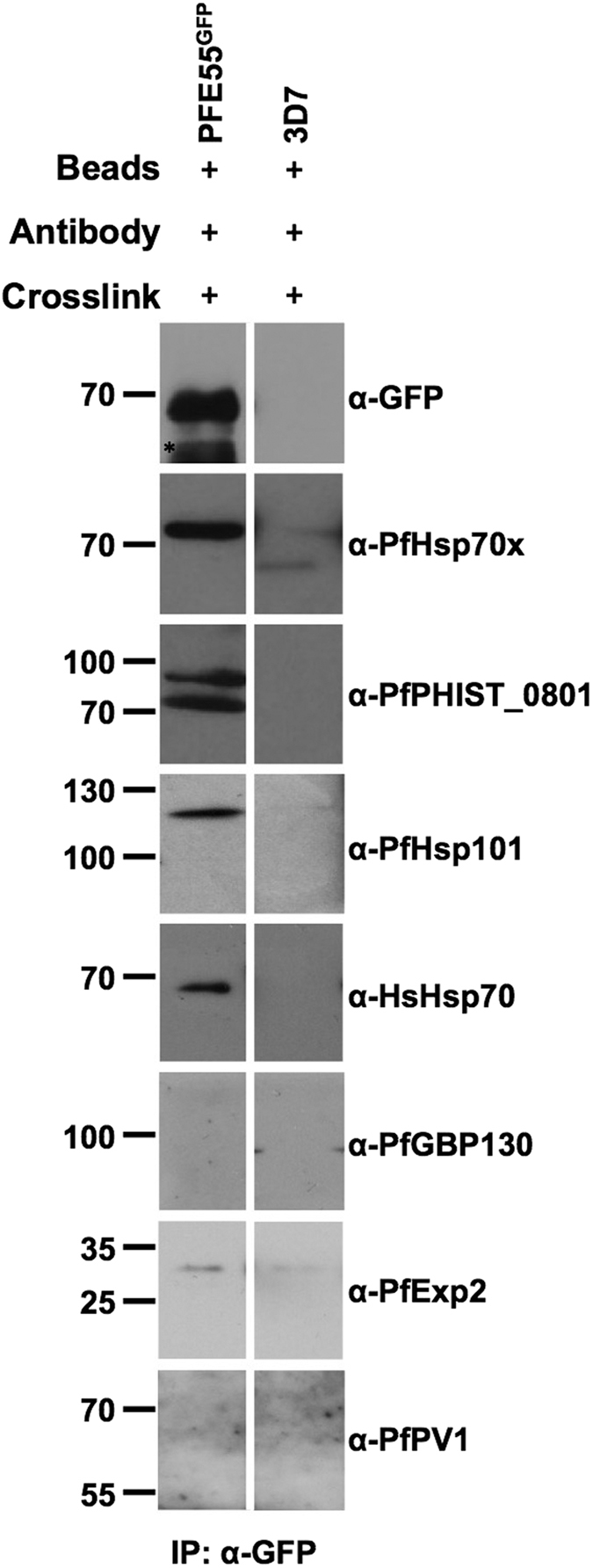
Immunodetection of anti-GFP co-IP verifies mass spectrometry data. Size markers in kDa. Antibodies used for immunodetection are indicated on right. Star indicates heavy chain of antibody used for co-IP. Full-length blots are presented in the Supplementary Information file.

This analysis reveals that PFE55 is incorporated into a complex or complexes containing PfHsp70x, PfPHIST_0801, PfHsp101 and HsHsp70 (Fig. 4). However, in contrast to complexes based around PfHsp70x, we could not identify either PfGBP130 or PfPV1 as being part of these complexes (Fig. 4). Although we could detect a small amount of PfExp2 in the immunoprecipitate generated from the PFE55^GFP^ lysate, a signal of a similar strength could also be found in the negative control (Fig. 4), suggesting that both signals are due to non-specific binding, and that PfExp2 is not likely to be a constituent of this complex.

### PfPHIST_0801 and PfGEXP18 are J-dots proteins, PfGEXP18 is incorporated into PfHsp70x-containing complexes

Our initial mass spectrometry analysis identified PfPHIST_0801 and PfGEXP18 as potential components of PfHsp70x/PFE55 containing complexes. For PfPHIST_0801 this could additionally be verified by crosslink and Immonoblotting (Figs 3 and 4). However, although both these proteins are predicted to be exported, their exact sub-cellular localization remains unknown.

**Figure 5:**
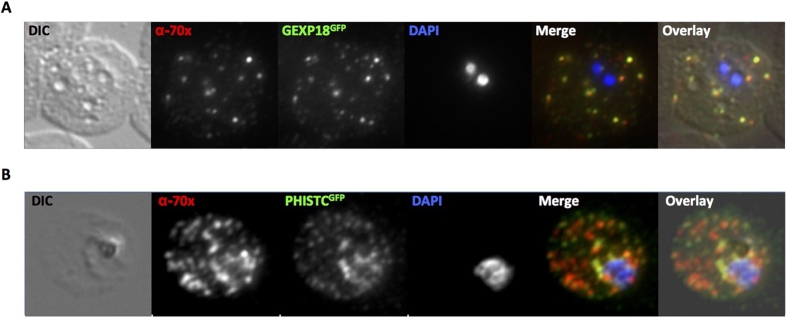
GEXP18 and PfPHIST_0801 are J-dot proteins. (**A**) Immunofluorescence using the J-dot marker PfHsp70x shows good colocalization with the GEXP18^GFP^ chimera. (**B**) Immunofluorescence using the J-dot marker PfHsp70x shows good colocalization with PfPHIST_0801^GFP^. 70x, PfHsp70x; DIC, differential interference contrast, PHISTC, PfPHIST_0801.

No specific antisera were available against PfGEXP18, so we generated a transgenic parasite line episomally expressing a C-terminal GFP tagged version of PfGEXP18. Fluorescence microscopy of human erythrocytes infected with this parasite line (referred to as GEXP18^GFP^) revealed a localization of the chimeric proteins to highly fluorescent foci within the infected cell (Fig. 5A). Live cell imaging demonstrated that these fluorescent foci were highly mobile (Supplementary Movie S3). As such a fluorescent pattern and mobility is highly indicative of a J-dot localization, we carried out co-immunofluorescence using antisera against the J-dot marker PfHsp70x. This analysis reveals a high level of signal colocalization in the infected erythrocyte (Fig. 5A), suggesting that PfGEXP18 is a *bona fide* J-dot protein.

Althgouh we were able to obtain specific antisera against PfPHIST_0801 (a kind gift of Brendan Crabb and Paul Gilson^12^), we were not able to use this for colocalization with PfHsp70x as both antibodies were raised in rabbit. Instead, we obtained a transgenic parasite lines expressing a PfPHIST_0801-GFP chimera (a kind gift of Hans-Peter Beck) and similarly carried out colocalization analysis with PfHsp70x. This experiment revealed good colocalization between PfPHIST_0801 and PfHsp70x (Fig. 5B) suggesting that PfPHIST_0801, as well as PfGEXP18, localizes to the J-dots.

**Figure 6:**
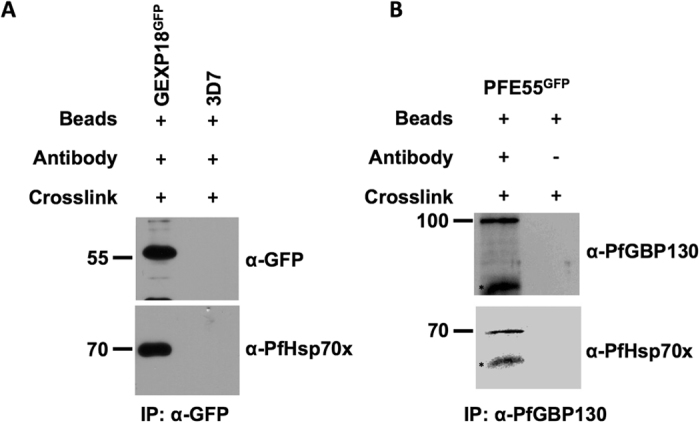
Reverse immunoprecipitation confirms an interaction between PfHsp70x, GEXP18 and PfGBP130. Cell lines used for co-IP are indicated above panels. Size markers in kDa. Antibodies used for immunodetection are indicated on right. Star indicates heavy chain of antibody used for co-IP. Full-length blots are presented in the Supplementary Information file.

Mass spectrometry suggested that PfGEXP18 is incorporated into a PfHsp70x complex. To corroborate this using an independent method, we crosslinked human cells infected with the GEXP18^GFP^ cell line, followed by immunoprecipitation using anti-GFP antibodies and immunoblotting using specific antisera. This analysis reveals that PfHsp70x and PfGEXP18 are found in a common protein complex (Fig. 6A). As PfGEXP18 is only found at the J-dots (Fig. 5A), this entire complex is likely to be localized to the J-dots. As we do not possess specific antisera raised against PFE55, we were unfortunately not able to investigate if this protein is also present in the complex. However, our mass spectrometry data suggest that PfGEXP18 is found only in complex with PfHsp70x and not PFE55 (Table 1), as peptides derived from PfGEXP18 were only found when immunoprecipitating using anti-PfHsp70x antisera.

### PfGBP130 is incorporated only into PfHsp70x-containing complexes, but is absent from PFE55 complexes

Both our mass spectrometry and immunoblot analyses suggested that, while PfGBP130 could be found in PfHsp70x-containing complexes, it was absent from complexes containing PFE55. To substantiate this conclusion, we crosslinked human erythrocytes infected with the PFE55^GFP^ cell line, followed by immunoprecipitation using anti-PfGBP130 specific antisera and immunoblotting using specific antisera. Immunoprecipitates probed with anti-PfHsp70x antisera verify that PfGBP130 and PfHsp70x are found in a common complex (Fig. 6B), however PFE55 appears to be absent from this complex (Fig. 4). Taken together with our previous data, this suggests that PfHsp70x is incorporated into at least two distinct multi-protein complexes.

### Interaction of PfHsp70x with PfHsp101 is sensitive to proteinase K after SLO treatment of infected cells

We were intrigued to find that PfHsp70x could be found in a complex with PfHsp101. PfHsp101 has previously been described as a component of the vacuolar protein translocon complex PTEX, and has been localized to the lumen of the PV^12^. PfHsp101 is predicted to be involved in the unfolding of protein substrates destined for transport to the host erythrocyte via the PTEX translocon. As a member of the ClpB family, the activity of PfHsp101 could potentially be regulated by interaction with an Hsp70 homologue^13^. As PfHsp70x is the only Hsp70 homologue so far identified in the lumen of the PV, we wished to understand how PfHsp101 and PfHsp70x interact. PfHsp70x localizes both to the host erythrocyte and the lumen of the PVM, thus we hypothesised that any complex including PfHsp101 and PfHsp70x must be found either in the host erythrocyte, or in the PV.

**Figure 7:**
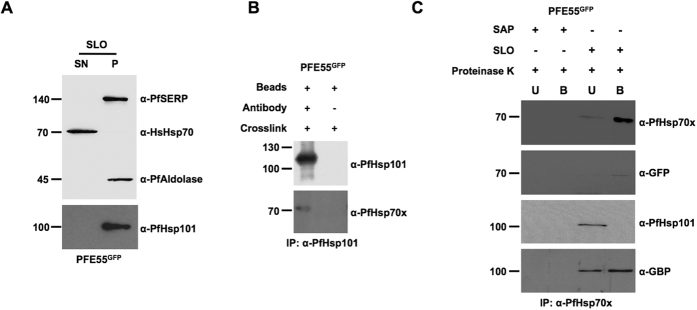
PfHsp101-PfHsp70x interaction takes place across the parasitophorous vacuolar membrane. (**A**) SLO permeabilisation verifies that PfHsp101 is restricted to the PV lumen. (**B**) Reverse immunoprecipitation verifies PfHsp101-PfHsp70x interaction. (**C**) PfHsp101-PfHsp70x interaction is protease sensitive following SLO permeabilisation. Size markers in kDa. Antibodies used for immunodetection are indicated on right. B, bound; U, unbound; P, pellet; S, supernatant. Full-length blots are presented in the Supplementary Information file.

Firstly, we verified that PfHsp101 is only found within the lumen of the PV. To this end, we carried out both immunofluorescence and cell fractionation experiments. Indirect immunofluorescence using antisera specific to PfHsp101 reveals a “ring” of fluorescence surrounding the body of the parasite, and no fluorescence within the host cell, consistent with a PV localization of this protein (Supplementary Fig. S4). We then fractionated infected cells using the bacterial pore-forming protein streptolysin O. This reagent permeabilises only the erythrocyte plasma membrane, leaving the PVM and parasite intact, and thus allows separation of the host cell cytosol from PV and parasite protein fractions. To control for correct fractionation, we detected the compartment-specific markers HsHsp70 (erythrocyte cytosol), PfSERP (PV lumen) and Pfaldolase (parasite cytosol). As expected, PfSERP and Pfaldolase were detected only in the pellet (PV and parasite) fraction, whereas HsHsp70 was present only in the supernatant (host cell cytosol) fraction. Analysis of the pellet and supernatant fraction reveals that PfHsp101 is only found in the pellet (PV/parasite) fraction, and cannot be detected in the host erythrocyte (supernatant fraction, Fig. 7A). Taken together these data verify that PfHsp101 is found only in the PV.

Secondly, we verified the presence and specificity of the PfHsp70x-PfHsp101 by reverse IP following crosslinking. We had already shown that PfHsp101 can be detected in PfHsp70x immunoprecipitates prepared from crosslinked cells (Fig. 3). We then used a specific monoclonal antibody to immunoprecipiate PfHsp101 complexes from crosslinked cells. This immunoprecipitate could be shown by immunoblot to contain PfHsp70x (Fig. 7B).

As PfHsp101 is only found in the PV, we thought it likely that any PfHsp70x/PfHsp101 complex should be found in this compartment. To experimentally interrogate this, we again permeabilised infected erythrocytes using streptolysin O, then added exogenous proteinase K, which would digest any residual protein complexes in the erythrocyte compartment (including J-dots), whilst leaving PV complexes intact^8^. Following this, we crosslinked protein complexes and carried out immunoprecipitation using anti-PfHsp70x antibodies. As a control, we carried out the same experiment, but substituted saponin for SLO. Saponin permeabilises both the erythrocyte plasma- and parasitophorous vacuolar- membranes, thus allowing access of proteinase K to the vacuolar contents. As expected, upon saponin and proteinase K treatment, no PfHsp70x could be immunoprecipitated. To our surprise, following treatment of SLO-permeabilised cells with proteinase K, although we could still precipitate PfHsp70x in complex with PfGBP130 and PFE55, PfHsp101 was no longer part of this complex (Fig. 7C). Thus, the complex containing PfHsp70x and PfHsp101 is protease sensitive.

To further investigate the protease sensitivity of PfHsp70x-containing complexes, we analysed immunoprecipitated PfHsp70x crosslink products by mass spectrometry. Here again, although we were still able to identify PfGBP130 and PFE55, PfHsp101 was no longer present (Table 2, Supplementary Table S5), supporting a model in which the PfHsp70x-PfHsp101 interaction is sensitive to protease treatment.

## Discussion

**Figure 8:**
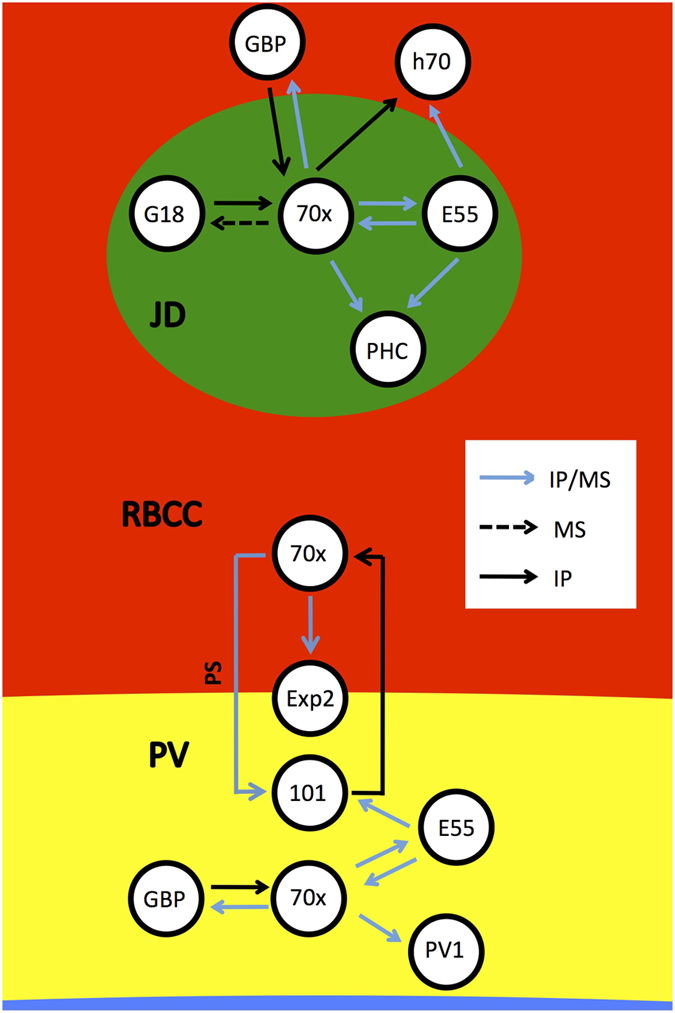
Model showing verified protein interactions identified and verified using various methods. JD, J-dot; RBCC, red blood cell cytosol; PV, parasitophorous vacuole; GBP, PfGBP130; h70, human Hsp70; G18, GEXP18; 70x, PfHsp70x; E55, PFE55; Exp2, PfExp2; 101, PfHsp101; PV1, PfPV1; PHC, PfPHIST_0801; IP, immunodetection following crosslink and immunoprecipitation; MS, mass spectrometry following crosslink and immunoprecipitation, PS, protease sensitive.

The malaria parasite massively changes the physical and biochemical properties of its chosen host cell, the mature human erythrocyte. These changes appear to be largely mediated by proteins that the parasite exports to the host cell^2^. We have previously reported that the parasite exports both co-chaperones of the Hsp40 family, and a single chaperone of the Hsp70 family to the host cell^8^,^10^. Whilst the Hsp40s (PFE55, PFA660) appear to be restricted to structures known as J-dots, the Hsp70 (PfHsp70x) is found both at the J-dots, and within the lumen of the parasitophorous vacuole^8^,^10^. To gain greater insight into the potential function of these proteins at their respective localizations, we have here used multiple proteomic and biochemical methods to identify and characterise protein complexes containing either the exported chaperone PfHsp70x or PfHsp40 cochaperone PFE55. An overview of our data is presented in Fig. 8.

Initially, we isolated an enriched J-dot fraction using differential centrifugation, and analysed complexes within such using blue native- and 2D Blue native/SDS-PAGE. This analysis suggested that both PfHsp70x and PFE55 can be found at the J-dots within at least four high molecular weight (HMW) complexes at >669, 440–669 and >232 kDa. Although we used a mild digitonin treatment to solubilise our samples prior to gel loading, we cannot fully exclude that the lower molecular weight complexes may represent breakdown products of the >669 kDa complex. Nevertheless, we are able to show that both PFE55 and PfHsp70x are incorporated into at least one HMW complex.

To try and elucidate further components of these, and other complexes, we carried out crosslinking on erythrocytes infected with transgenic parasites expressing GFP tagged PFE55 (PFE55^GFP^), immunoprecipitated using either anti-GFP or anti-PfHsp70x specific antisera, and analysed the resulting protein fraction by mass spectrometry and immonoblotting. This allowed us to identify a large number of candidate proteins that may be present in complexes containing PfHsp70x, PFE55, or both. After prioritisation according to predicted subcellular localization and peptide count, we decided to study several interactions in detail using either specific antisera or transgenic parasite lines. We find that, similar to our mass spectrometry results, PfHsp70x appears to interact with a larger number of proteins than does the co-chaperone Hsp40. Indeed, apart from the interaction with PfHsp70x itself, PFE55 only appears to interact with 5 further proteins. This is not unexpected; most genomes contain a larger number of Hsp40 then Hsp70 homologues, and indeed the large contingent of Hsp40 proteins appears to be required for recruitment of cellular Hsp70s to specific substrates, in effect acting as a molecular adapter allowing a small number of Hsp70s to act on a larger number of substrate proteins^14^. Here we have studied only one specific exported Hsp40, PFE55, but the parasite is predicted to export 19 Hsp40 homologues to the host erythrocyte, where each individual homologue may recognise a specific sub-set of protein substrates^15^.

Both PfHsp70x and PFE55 seem to be found in a complex with the PHISTc family member encoded by PF3D7_0801000 (referred to as PfPHIST_0801). PHIST (Poly-Helical Interspersed Sub-Telomeric) proteins are encoded by a large multi-gene family, with 72 members in *P. falciparum*. The family is further split into three sub-families (PHISTa, b, c) based on species distribution^6^. Several PHIST proteins have been localized within the infected cell, and have been associated with the erythrocyte membrane, knobs and the erythrocyte cytoskeleton^16^,^17^,^18^,^19^,^20^. It was recently suggested that one function of PHIST proteins could be to assist in either the transport or presentation of PfEMP1 to/on the surface of the infected cell^19^. Using specific anti-sera, we were able to demonstrate that PfPHIST_0801 is a J-dot protein. Furthermore, our mass spectrometry data indicates that PfHsp70x also interacts with two further PHIST family members, one of which has been previously localized to the erythrocyte cytosol^16^. We have previously suggested that one role of PfHsp70x, and indeed of the J-dots themselves may be to aid in transport of PfEMP1 through the host cell, and the discovery of PHIST proteins at the J-dots adds weight to this idea. Our data is consistent with a model in which PfHsp70x together with exported-co-chaperones such as PFE55 are involved, in conjunction with members of the PHIST family, in chaperoning PfEMP1 in a soluble state through the host cell cytosol before insertion into the Maurer’s clefts or knobs. Notably, we did not detect PfEMP1 in any of our co-immunoprecipitation experiments, despite our use of crosslinkers to stabilize intermolecular interactions. We wish to note that PfEMP1 is a protein of high molecular weight, low abundance and unusual solubility characteristics and may have therefore been lost during sample preparation. We cannot thus exclude that PfEMP1 may be part of the PfHsp70x/PFE55/PfPHIST_0801 complex. Moreover, we have previously shown that PfEMP1 colocalizes with PFE55 during export to the host cell surface, suggesting a functional interaction^8^.

PfGEXP18 was also identified as a potential interaction partner of PfHsp70x. This protein was originally described in a proteomics analysis as being an exported protein of early sexual gametocyte stages^21^. However, expression and proteomics data suggest that PfGEXP18 is also expressed in asexual blood stage parasites^22^. As the localization of this protein was not known, and no specific antisera were available, we generated transgenic parasites expressing a PfGEXP18-GFP chimera, could localize this chimera to mobile J-dots and could demonstrate incorporation of the chimera into a complex containing PfHsp70x. There is no known function for PfGEXP18, and therefore we are unable to say whether this protein is incorporated into the complex as part of the trafficking machinery, or as a substrate being trafficked. Nevertheless, our study has verified at least two further J-dot proteins, and generated further candidates whose localization and function can be investigated in future studies.

PfHsp70x, as previously mentioned, is found at the J-dots, but also within the lumen of the PV. We were therefore interested to note a potential interaction between PfHsp70x and PfHsp101, a component of the PTEX translocon within the PVM^12^. We, and others, have previously suggested that PfHsp70x may interact with the translocon, on either the *cis*- or *trans*- side of the PVM^10^,^23^. To study this interaction in further detail, we used differential cell lysis followed by proteinase K treatment to remove PfHsp70x present in the erythrocyte cytosol (including J-dots). To our surprise, this treatment also led to breakdown of the PfHsp70x-PfHsp101 complex. As the contents of PV lumen, and thus PfHsp101, should be protected from proteinase K, this forces us to conclude that the PfHsp70x-PfHsp101 complex must span the PVM, with only PfHsp70x being susceptible to proteolytic degradation. We interpret this data as suggesting that the PfHsp70x-PfHsp101 interaction is indirect, and likely mediated by further proteinaceous factors that cross the PVM. One protein of particular interest in this light is PfExp2. PfExp2 is hypothesized to form pores within the PVM through which unfolded proteins may be translocated to reach the erythrocyte, and is therefore an important component of the PTEX translocon^12^. Our mass spectrometry and Immonoblot data indicate that PfExp2 is incorporated into a complex together with PfHsp70x and PfHsp101, and thus fulfills the criteria to be the membrane crossing component of the complex, possibly forming a bridge between PfHsp101 (in the PV lumen), and PfHsp70x (in the erythrocyte cytosol). However, even following proteinase K treatment, we were still able to detect an interaction of PfHsp70x with PfGBP130 and (albeit weak) PFE55, implying that these proteins are incorporated into a common complex within the lumen of the PV. This complex is independent of the PfHsp101-PfHsp70x complex described above. Whilst it is known that PfGBP130 is dually localized to both the erythrocyte cytosol and PV, until this point PFE55 has been detected only at the J-dots^8^,^24^. Our most recent data therefore suggests that PFE55 is also present (likely in low abundance) within the lumen of the PV, where it may act as a co-chaperone for PV localized PfHsp70x. As PfGBP130 is also exported to the erythrocyte cytosol, we are unable to exclude that this protein is also incorporated into further PfHsp70x-containing complexes within the host erythrocyte.

A very recent study has suggested that both PfPV1 and PfHsp70x are associated with the PTEX translocon complex^23^. Our data add additional support for this model, as we were able to identify PfHsp70x in complex with the PTEX component PfHsp101. Additionally, we identified PfPV1 as a potential interactor of PfHsp70x, although we are not able to demonstrate if this interaction is direct, or possibly mediated by additional proteins. Elsworth, Sanders *et al*. suggest that PfHsp70x may associate with PTEX on the trans- side of the PVM, and may act to receive protein cargo passing through the translocon^23^. Here we present the first direct experimental evidence in support of this interpretation, by demonstrating that the PfHsp70x-PfHsp101 interaction takes place across the PVM. However, the fact that PfHsp70x is found within the PV lumen in complex with PfGBP130 and PFE55 suggests that it may also have an important function in this compartment.

Although not the focus of this current study, we were interested to find human Hsp70 (HsHsp70) in complex with PFE55. Before the discovery of the exported parasite chaperone PfHsp70x, it was long believed that residual human Hsp70 in the erythrocyte may be undergoing interactions with exported parasite Hsp40 proteins^8^. In this model, parasite-encoded Hsp40s modulate the function of human Hsp70 and allow it to use exported parasite proteins as substrates^25^. Functional support for this model is so far lacking, and indeed a recent study suggests that the exported parasite Hsp40 PFA660 acts as a specific co-chaperone for PfHsp70x, but does not functionally interact with human Hsp70^26^. Nevertheless, a potential interaction of human Hsp70 with the PTEX translocon has also been described, and our data also support a role for human Hsp70 in the infected erythrocyte^12^. Further studies will be necessary to understand the significance of this result in relation to protein traffic and parasite survival.

The *Plasmodium falciparum*-infected erythrocyte is a complex compartment containing, as well as the *circa* 1900 human proteins, over 400 parasite-encoded proteins, and several novel parasite-induced structures such as Maurer’s clefts and tethers, the tubulovesicular membrane network and the less well characterized J- and K- dots^4^,^5^,^6^,^8^,^9^,^27^. Infected erythrocytes have different biochemical and physical properties to their non-infected counterparts, and it is generally accepted that parasite-encoded proteins are responsible for many of these modifications^2^,^28^. However, our knowledge of how exported parasite proteins interact with each other, and with parasite-induced structures to bring about these changes is still basic. Our laboratory is interested in how exported proteins are involved in host cell modification, specifically the transport of parasite-encoded proteins to the host cell plasma membrane. We have previously implicated exported Hsp40s and a single exported Hsp70 in this process, and have here begun to unravel their molecular interactions to gain information on their integration into potential functional protein networks^8^,^10^. We are able to identify and characterize several protein complexes with different cellular localizations, and our data suggest that particularly PfHsp70x is involved in a multitude of protein-protein interactions, and therefore may represent an important hub to coordinate protein trafficking processes. As PfHsp70x is the only exported Hsp70 found in the infected erythrocyte, small molecules capable of interfering with the activity of this protein have the potential to interrupt multiple processes at once, many of which may be essential for parasite survival^29^,^30^,^31^.

## Materials and Methods

### Differential centrifugation

Erythrocytes infected with trophozoite stage parasites were lysed in 5 mM Na_2_HPO_4_ buffer containing protease inhibitor cocktail (Roche), 1 mM PMSF and fractionated by differential centrifugation at 4 °C; 1000 *g*, 10 min; 5000 *g*, 10 min; 10000 *g*, 10 min; 20000 *g*, 10 min; 33000 g, 30 min; 80000 *g*, 2 h; 100,000 *g*, 2 h. Each pellet was washed twice with lysis buffer. Pellets were either resuspended in SDS-PAGE sample buffer (for immunodetection), or prepared for BN-PAGE as described below.

### BN-PAGE and 2D BN/SDS-PAGE

Blue native PAGE gels were prepared and run as previously described^32^,^33^. Briefly, the final pellet resulting from differential centrifugation was resuspended in BN sample buffer (20 mM bis-tris, 500 mM 6-aminocaproic acid, 20 mM NaCl, 2 mM pH 8.0 EDTA, 10% Glycerol, pH 7.0) with 0.5% digitonin and 0.01% Coomassie Brilliant G-250, and separated on a 4% stacking gel and 4–15% gradient separating gel. High molecular weight (HMW) Native markers were used as a standard (GE Healthcare). Proteins were then either transferred to nitrocellulose membrane for immunodetection, or used in second dimension separation; the first dimension BN-gel was sliced and boiled in SDS sample buffer for 20 s followed by incubation on a shaker (RT, 30 min). The denatured gel slice was then inserted into the well of a 10% SDS-PAGE gel and separated. Proteins were transferred to nitrocellulose for immunodetection.

### *In vivo* crosslink and co-immunoprecipitation

Erythrocytes infected with trophozoite stage parasites were enriched by Gelafundin flotation and washed twice in PBS (pH 7.2), followed by addition of DSP to 1 mM. The reaction was allowed to proceed on ice for 2 h and was then terminated by addition of Tris-HCl to 15 mM and incubation at room temperature for a further 15 min. Crosslinked cells were lysed with IP buffer (IP, Pierce co- immunoprecipitation kit, Thermo Scientific, Rockford, IL, USA) and centrifuged to remove cellular debris (4 °C, 1300 *g*). The supernatant was used to perform immunoprecipitation using monoclonal mouse anti-GFP (Roche), polyclonal rabbit anti-PfHsp70x^10^, monoclonal mouse anti-PfHsp101^12^ or polyclonal rabbit anti-PfGBP 130^24^. Proteins were eluted with 0.1% TFA, and separated by 12% SDS-PAGE followed by either in-gel trypsin digestion or transfer to nitrocellulose followed by immunodetection.

### Sample Preparation for LC-MS/MS and data analysis

SDS gels were stained with colloidal coomassie to visualize protein bands. Gel bands were washed in milli-Q water and cut into 1 mm^3^ pieces, followed by reduction (10 mM DTT, 56 °C, 1 h), alkylation (55 mM IAA, RT, 45 min in the dark) and digest with trypsin (Promega, 1:30 w/w, 37 °C overnight). Digested peptides were concentrated and analyzed via LTQ-Orbitrap Elite Mass spectrometry (Thermo-Fisher, Waltham, MA, US) equipped with an EASY-Spray source and nano-LC UltiMate 3000 high-performance liquid chromatography system (Thermo-Fisher, Waltham, MA, US).

### Experimental Design and Statistical Rationale

A full-scan survey MS experiment (m/z range from 375 to 1600; automatic gain control target, 1,000,000 ions; resolution at 400 m/z, 60,000; maximum ion accumulation time, 50 ms) was performed using the Orbitrap mass spectrometer, and the ten most intense ions were fragmented by collision-induced dissociation (CID). Raw data were converted to mgf files by Proteome Discoverer 1, and then identified using pFind 2.1 software to search in the human Uniprot_TrEMBL database (release on April 2012, human, 65,493 entries) and PlasmoDB database (release on October 2013, *Plasmodium falciparum* 3D7), respectively^22^,^34^. Modifications were set as follows: static modification of carbamidomethyl (Cys), dynamic modification of deamination (Asn), oxidation (Met), and acetylation (Lys). Trypsin was selected as the enzyme, and two missed cleavages were allowed. The mass tolerance was set to 20 ppm for the precursor ions and 0.5 Da for the fragment ions. A false discovery rate (FDR) of 1% was estimated and applied to all data sets at the total peptide level. The mass spectrometry proteomics data have been deposited to the ProteomeXchange Consortium (http://proteomecentral.proteomexchange.org) via the PRIDE partner repository with the dataset identifier PXD003789.

### Antibodies for immunodetection and immunoprecipitation

Antibodies for immunodetection: Monoclonal mouse anti-GFP (Roche, 1:1000); polyclonal rabbit anti-PfHsp70x (1:500^10^); polyclonal rabbit anti-PfSERP (1:1000^24^); polyclonal rabbit anti-Pfaldolase (1: 5000^24^); polyclonal rabbit anti-PfSBP1 (BR5, 1:500^35^); polyclonal rabbit anti-Exp1 (1:500^36^); monoclonal mouse anti-glycophorin A/B (Sigma, 1:1000); polyclonal rabbit anti-GBP130 (1:1000^24^); monoclonal mouse anti-Hsp101 (1:1000^12^); polyclonal rabbit anti-PfPHIST_0801 (1:1000^12^); monoclonal mouse anti-HsHsp70 (Santa Cruz SC24, 1:500); monoclonal mouse anti-PfEXP2 (1:500^12^); polyclonal rabbit anti-PfPV1 (1:500^37^). All antibodies were diluted in 5% skimmed milk powder/PBS. Secondary antibodies were from Dianova (Hamburg, 1:2000).

### Antibodies for immunofluorescence

Polyclonal rabbit anti-PfHsp70x (1:500^10^); polyclonal rabbit anti-PfPHIST_0801 (1:1000^12^); polyclonal chicken anti-GFP (Abcam, 1:100). All antibodies were diluted in 3% BSA/PBS. Secondary antibodies (Jackson, Cy2, Cy3) were diluted 1:2000.

### Parasite cell culture and transgenic parasite lines

*Plasmodium falciparum* parasite (clone 3D7 and transfectant lines) were cultured as previously described^38^ in RPMI media (Gibco) supplemented with 5% human serum, 0.25% albumax II (Invitrogen), 200 μM hypoxanthine and 150 μM neomycin. The PFE55^GFP^ transfectant line has been previously described^8^. To generate parasites expressing a GFP tagged version of PfGEXP18, the full length coding sequence of *Pfgexp18* (PlasmoDB: PF3D7_0402400) was amplified without the stop-codon from total RNA (*P. falciparum* 3D7) using the SuperScriptII One Step RT-PCR (Invitrogen) using primers PF3D7_0402400_XhoI_F (AATCTCGAGATGATATATATAAGGAAGGATAAATTACG) and PF3D7_0402400_AvrII_R (ATTCCTAGGTTCTACTACAGAATCTGCTGTGTTAAAATC). The XhoI/AvrII restricted PCR product was inserted into pARL2-GFP^39^. The construct was verified by restriction digest and DNA sequencing (GATC Biotech). Transfection was conducted at a parasitemia of approximately 10% ring stages (3D7) with 100 μg of plasmid DNA in fresh 0^+^ human erythrocytes under standard conditions^40^. The transfectants were selected using 2 nM WR99210 (Jackson Pharmaceuticals). After 31 days transfectant parasites reappeared.

### Live cell imaging and immunofluorescence

All immunofluorescence assays were performed with parasite smears fixed for 10 min in −20 °C acetone/methanol (90%/10%) as previously described^8^. Nuclear DNA was visualised with DAPI. Images were taken using a Zeiss Axio Observer inverse epifluorescence microscope.

### Image processing and presentation

Individual images were imported into Image J64 (version 1.48b, available at http://rsb.info.nih.gov/ij) and converted to 8-bit greyscale. To create figures, TIF files were imported into PowerPoint (Microsoft), assembled and slides exported as TIFs. No gamma adjustments were applied to any images, and all data are presented in accordance with the recommendations of Rossner and Yamada^41^.

### Streptolysin O (SLO) permeabilization

Erythrocytes infected with trophozoite stage parasites were enriched by Gelafundin flotation and washed twice in PBS (pH 7.4). Aliquots of 2 × 10^8^ cells were then subjected to 3 hemolytic units of SLO in 188 μl of PBS (pH 7.4) and incubated for 6 min at RT with gentle mixing every 2 min, followed by centrifugation at (1000 *g*, 3 min). The supernant was mixed with 2x SDS loading buffer and boiled for 10 min. The resulting pellet was either used for proteinase K protection (see below), or mixed with SDS loading buffer and boiled for 10 min.

### Saponin permeabilization

Erythrocytes infected with trophozoite stage parasites were enriched by Gelafundin flotation, washed twice in PBS (pH 7.4), treated with 0.02% saponin/PBS (pH 7.4) on ice for 10 min with gentle mixing, then separated into pellet and supernatant fractions. The pellet was washed twice in PBS and used for the proteinase K protection assay (see below).

### Proteinase K protection assay

The pellet fraction from SLO or saponin permeabilization was incubated with 0.5 mg/ml proteinase K (Carl Roth) on ice for 30 min. To stop the reaction, 2 mM PMSF and protease inhibitor cocktail were added. The pellet was then collected by centrifugation and used for subsequent crosslink and immunoprecipitation.

## Additional Information

**How to cite this article:** Zhang, Q. *et al*. Proteomic analysis of exported chaperone/co-chaperone complexes of *P. falciparum* reveals an array of complex protein-protein interactions. *Sci. Rep.* **7**, 42188; doi: 10.1038/srep42188 (2017).

**Publisher's note:** Springer Nature remains neutral with regard to jurisdictional claims in published maps and institutional affiliations.

## Supplementary Material

Supplementary Movie 1

Supplementary Information

Supplementary Dataset 1

Supplementary Dataset 2

Supplementary Dataset 3

## Figures and Tables

**Table 1:** Selected proteins identified by crosslink followed by co-IP using either anti-GFP or anti-PfHsp70x.

Co-IP PFE55-GFP N = 7								
ID	Description	UP	PC	UP control	PC control	Ratio IP/C	Previous ID(s)	Localisation
*Exported N = 5*								
PF3D7_0501100.1/2	*heat shock protein 40, type II (HSP40)*	6	15,42	0	0,00	6/0	MAL5P1.12, PFE0055c	J-dots
PF3D7_0801000	*Plasmodium exported protein (PHISTc, PfPHIST_0801), unknown function*	3	4,10	0	0,00	3/0	PF08_0137	Unknown
PF3D7_1401100	DnaJ protein, putative	8	22,56	1	1,83	8,00	PF14_0013	Unknown
PF3D7_1407800	plasmepsin IV (PM4)	3	12,70	1	4,68	3,00	PF14_0075	FV
PF3D7_0831700	*heat shock protein 70 (HSP70-x)*	17	34,87	6	15,46	2,83	MAL7P1.228	J-dots
*Signal peptide N = 1*								
PF3D7_1116800	*heat shock protein 101 (HSP101)*	5	9,27	0	0,00	5/0	PF11_0175	PV/PVM
*TM Domain N = 1*								
PF3D7_1408100	plasmepsin III (HAP)	2	5,97	0	0,00	2/0	PF14_0078	FV
**Co-IP PfHsp70x N = 34**								
**ID**	**Description**	**UP**	**PC**	**UP control**	**PC control**	**Ratio IP/C**		
*Exported N = 9*								
PF3D7_0831700	*heat shock protein 70 (HSP70-x)*	38	57,44	3	12,08	12,67	MAL7P1.228	J-dots
PF3D7_0402400	*Plasmodium exported protein, unknown function (GEXP18)*	6	36,61	0	0,00	6/0	MAL4P1.23, PFD0115c	Unknown
PF3D7_1016300	*glycophorin binding protein (GBP)*	6	46,48	0	0,00	6/0	PF10_0159	PV/RBCC
PF3D7_0501100.1/2	*heat shock protein 40, type II (HSP40)*	4	15,92	0	0,00	4/0	MAL5P1.12, PFE0055c	J-dots
PF3D7_1201000	Plasmodium exported protein (PHISTb), unknown function	4	6,28	0	0,00	4/0	2277.t00010, MAL12P1.10, PFL0050c	Unknown
PF3D7_0401800	Plasmodium exported protein (PHISTb), unknown function (PfD80)	2	4,46	0	0,00	2/0	MAL4P1.16, PFD0080c	Erythrocyte periphery/cytoskeleton
PF3D7_0721100	conserved Plasmodium protein, unknown function	2	9,84	0	0,00	2/0	PF07_0087	Unknown
PF3D7_1001400	alpha/beta hydrolase, putative	2	3,47	0	0,00	2/0	PF10_0018	Unknown
PF3D7_0801000	*Plasmodium exported protein (PHISTc, PfPHIST_0801), unknown function*	9	12,22	0	0,00	9/0	PF08_0137	Unknown
*Signal peptide N = 18*								
PF3D7_1252100	rhoptry neck protein 3 (RON3)	9	5,60	0	0,00	9/0	2277.t00499, MAL12P1.496, PFL2505c	Rhoptry neck
PF3D7_1012200	conserved Plasmodium protein, unknown function	5	24,72	0	0,00	5/0	PF10_0119	Apical
PF3D7_1116800	*heat shock protein 101 (HSP101)*	4	6,51	0	0,00	4/0	PF11_0175	PV/PVM
PF3D7_1105800	conserved Plasmodium protein, unknown function	3	13,53	0	0,00	3/0	PF11_0069	Unknown
PF3D7_0302500	cytoadherence linked asexual protein 3.1 (CLAG3.1)	2	3,25	0	0,00	2/0	MAL3P1.5, PFC0120w	Apical/rhoptry
PF3D7_1015200.1/2	cysteine–tRNA ligase, putative (CysRS)	2	3,40	0	0,00	2/0	PF10_0149	Apicoplast
PF3D7_1116000	rhoptry neck protein 4 (RON4)	2	1,67	0	0,00	2/0	PF11_0168	Rhoptry neck
PF3D7_0930300	merozoite surface protein 1 (MSP1)	11	8,37	1	2,85	11,00	PFI1475w	Merozoite/ring surface
PF3D7_0207600	serine repeat antigen 5 (SERA5)	8	15,15	1	1,81	8,00	PF02_0072, PFB0340c	PV
PF3D7_1232100	60 kDa chaperonin (CPN60)	8	19,36	1	1,67	8,00	2277.t00309, MAL12P1.309, PFL1545c	Apicoplast
PF3D7_0827900	protein disulfide isomerase (PDI8)	5	18,63	1	3,42	5,00	MAL8P1.17	ER
PF3D7_0917900	heat shock protein 70 (HSP70-2)	19	36,35	4	7,36	4,75	PFI0875w	ER
PF3D7_0929400	high molecular weight rhoptry protein 2 (RhopH2)	25	25,04	6	6,39	4,17	PFI1445w	Rhoptry
PF3D7_1471100	exported protein 2 (EXP2)	4	14,98	1	3,83	4,00	PF14_0678	PVM
PF3D7_1222300	endoplasmin, putative (GRP94)	7	12,42	2	3,41	3,50	2277.t00214, MAL12P1.214, PFL1070c	ER
PF3D7_0905400	high molecular weight rhoptry protein 3 (RhopH3)	11	19,84	4	6,24	2,75	PFI0265c	Rhoptry
PF3D7_1129100	parasitophorous vacuolar protein 1 (PV1)	5	14,82	2	5,75	2,50	PF11_0302	PV
PF3D7_1454400	aminopeptidase P (APP)	17	29,09	8	13,26	2,13	PF14_0517	PV/FV
*TM Domain N = 7*								
PF3D7_0811200	ER membrane protein complex subunit 1, putative (EMC1)	4	5,21	0	0,00	4/0	MAL8P1.105	J-dots
PF3D7_0220000	liver stage antigen 3 (LSA3)	3	2,95	0	0,00	3/0	PF02_0187, PFB0915w	Unknown
PF3D7_0823800	DnaJ protein, putative	3	8,40	0	0,00	3/0	PF08_0032	PV/RBCC
PF3D7_1037300	ADP/ATP transporter on adenylate translocase (ADT)	3	15,62	0	0,00	3/0	PF10_0366	J-dots
PF3D7_1105800	conserved Plasmodium protein, unknown function	3	13,53	0	0,00	3/0	PF11_0069	Unknown
PF3D7_1459400	conserved Plasmodium protein, unknown function	2	7,94	0	0,00	2/0	PF14_0567	Unknown
PF3D7_1311800	M1-family alanyl aminopeptidase (M1AAP)	12	17,51	1	2,58	12,00	MAL13P1.56	Unknown
**Common to Co-IP GFP/PfHsp70x N = 4**								
**ID**	**Description**							
PF3D7_0501100.1/2	heat shock protein 40, type II (HSP40)							
PF3D7_0801000	Plasmodium exported protein (PHISTc, PfPHIST_0801), unknown function							
PF3D7_0831700	heat shock protein 70 (HSP70-x)							
PF3D7_1116800	heat shock protein 101 (HSP101)							

ID, PlasmoDB accession number; UP, unique peptides; PC, peptide coverage. Localization based on literature or Apiloc predictions. Proteins in italic were chosen for further study.

**Table 2:** Selected proteins detected following SLO permeabilisation, proteinase K treatment and co-IP using anti-PfHsp70x.

Co-IP PfHsp70x			
ID	Description	UP	PC
*Exported N = 3*			
PF3D7_0831700	heat shock protein 70 (HSP70-x)	41	55,523
PF3D7_1016300	glycophorin binding protein (GBP)	5	42,961
PF3D7_0501100.1/2	heat shock protein 40, type II (HSP40)	5	12,935
*Signal peptide N = 12*			
PF3D7_0930300	merozoite surface protein 1 (MSP1)	4	2,384
PF3D7_1232100	60 kDa chaperonin (CPN60)	3	5,989
PF3D7_0827900	protein disulfide isomerase (PDI8)	3	8,075
PF3D7_0917900	heat shock protein 70 (HSP70-2)	15	26,994
PF3D7_0929400	high molecular weight rhoptry protein 2 (RhopH2)	3	3,193
PF3D7_1222300	endoplasmin, putative (GRP94)	4	5,725
PF3D7_0905400	high molecular weight rhoptry protein 3 (RhopH3)	3	4,571
PF3D7_1454400	aminopeptidase P (APP)	15	26,255
PF3D7_1324900	L-lactate dehydrogenase (LDH)	7	29,114
PF3D7_1222300	endoplasmin, putative (GRP94)	4	5,725
PF3D7_0827900	protein disulfide isomerase (PDI8)	3	8,075
PF3D7_1410400	rhoptry-associated protein 1 (RAP1)	3	5,754
*TM Domain N = 1*			
PF3D7_1311800	M1-family alanyl aminopeptidase (M1AAP)	6	8,111

ID, PlasmoDB accession number; UP, unique peptides; PC, peptide coverage.
