# Supplementary material for: Proteomic analysis of exported chaperone/co-chaperone complexes of P. falciparum reveals an array of complex protein-protein interactions
Source: Sci Rep. 2017 Feb 20;7:42188. doi: 10.1038/srep42188 (PMC5316994; doi:10.1038/srep42188)
Supplement: Supplementary Information [file srep42188-s2.pdf]

Proteomic analysis of exported chaperone/co-chaperone complexes of *P. falciparum* reveals an array of complex protein-protein interactions.

Qi Zhang, Cheng Ma, Alexander Oberli, Astrid Zinz, Sonja Engels and Jude M Przyborski*

Legends to Supplementary Material

Supplementary Table S1. Proteins identified by crosslink followed by co-IP using either anti-GFP or anti-PfHsp70x. ID, PlasmoDB accession number; UP, unique peptides; PC, peptide coverage. Localization based on literature or Apiloc predictions.

Supplementary Table S2. Human proteins identified by crosslink followed by co-IP using either anti-GFP or anti-PfHsp70x. ID, Uniprot accession number; UP, unique peptides; PC, peptide coverage.

Supplementary Movie S3. **Live cell imaging of GEXP18^{GFP} cell line.** Highly mobile fluorescent foci can be observed within the infected erythrocyte.

Supplementary Figure S4. PfHsp101 localizes only to a "ring" surrounding the parasite, suggestive of a PV localization. DIC, differential interference contrast.

Supplementary Table S5. Total proteins detected following SLO permeabilisation, proteinase K treatment and co-IP using anti-PfHsp70x. ID, PlasmoDB accession number; UP, unique peptides; PC, peptide coverage.

Supplementary File S6. Full-length blots used to generate figures 1, 2, 3, 4, 6 and 7.

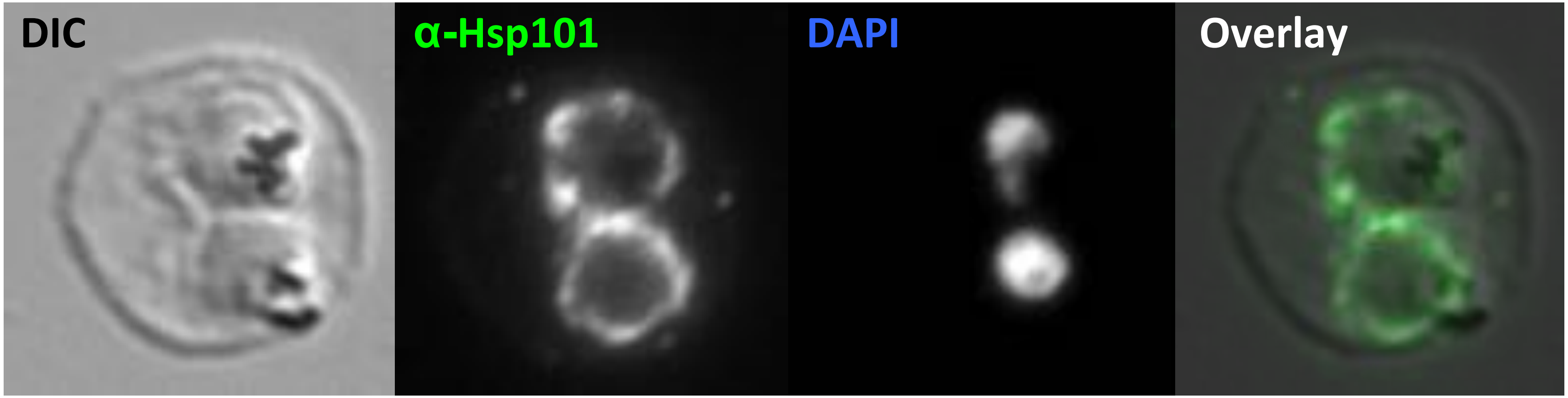

Supplementary Figure S4

Supplementary Information file S6

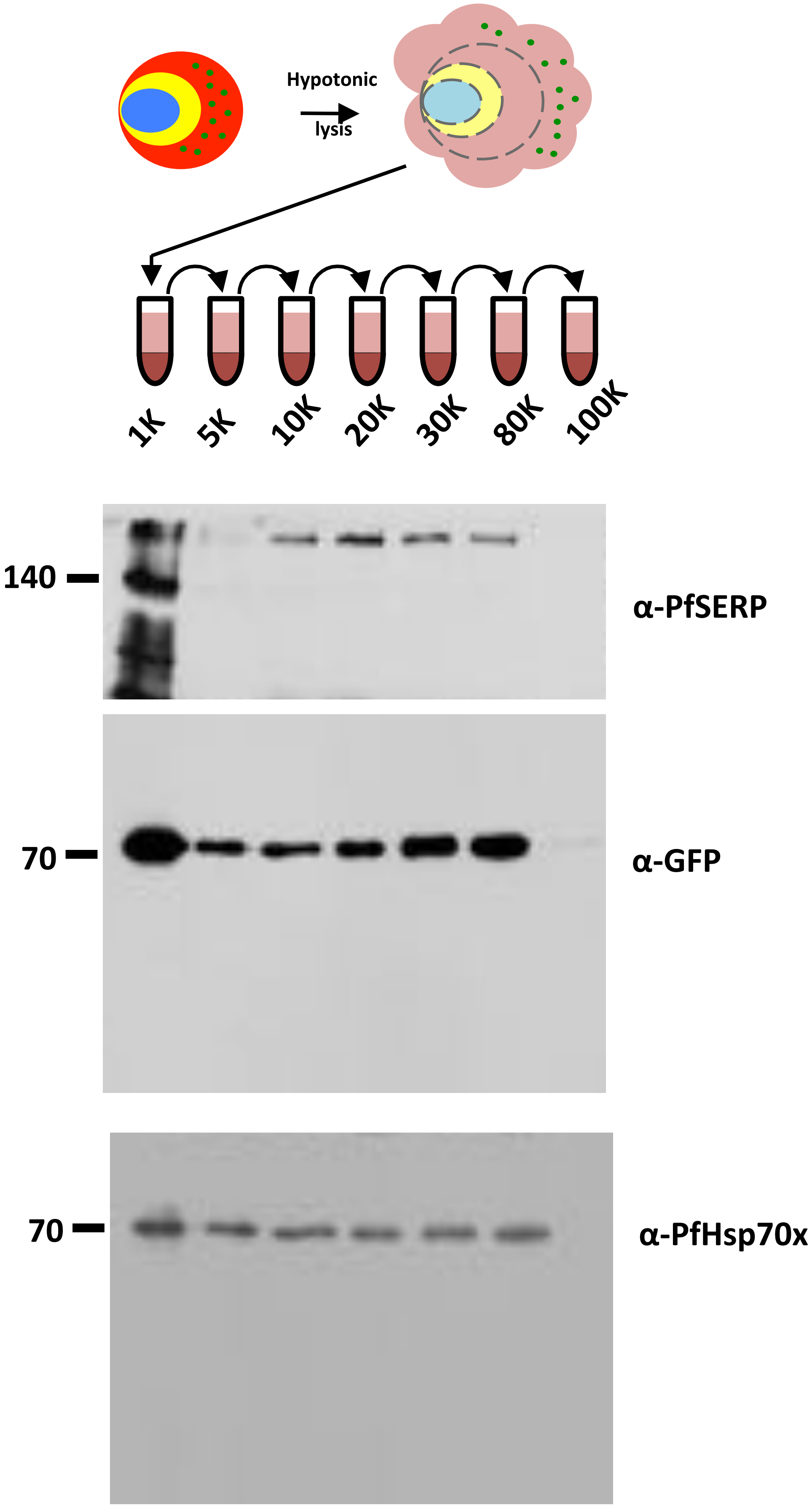
Hypotonic
lysis
1K
5K
10K
20K
30K
80K
100K
140
α-PfSERP
70
α-GFP
70
α-PfHsp70x

Figure 1

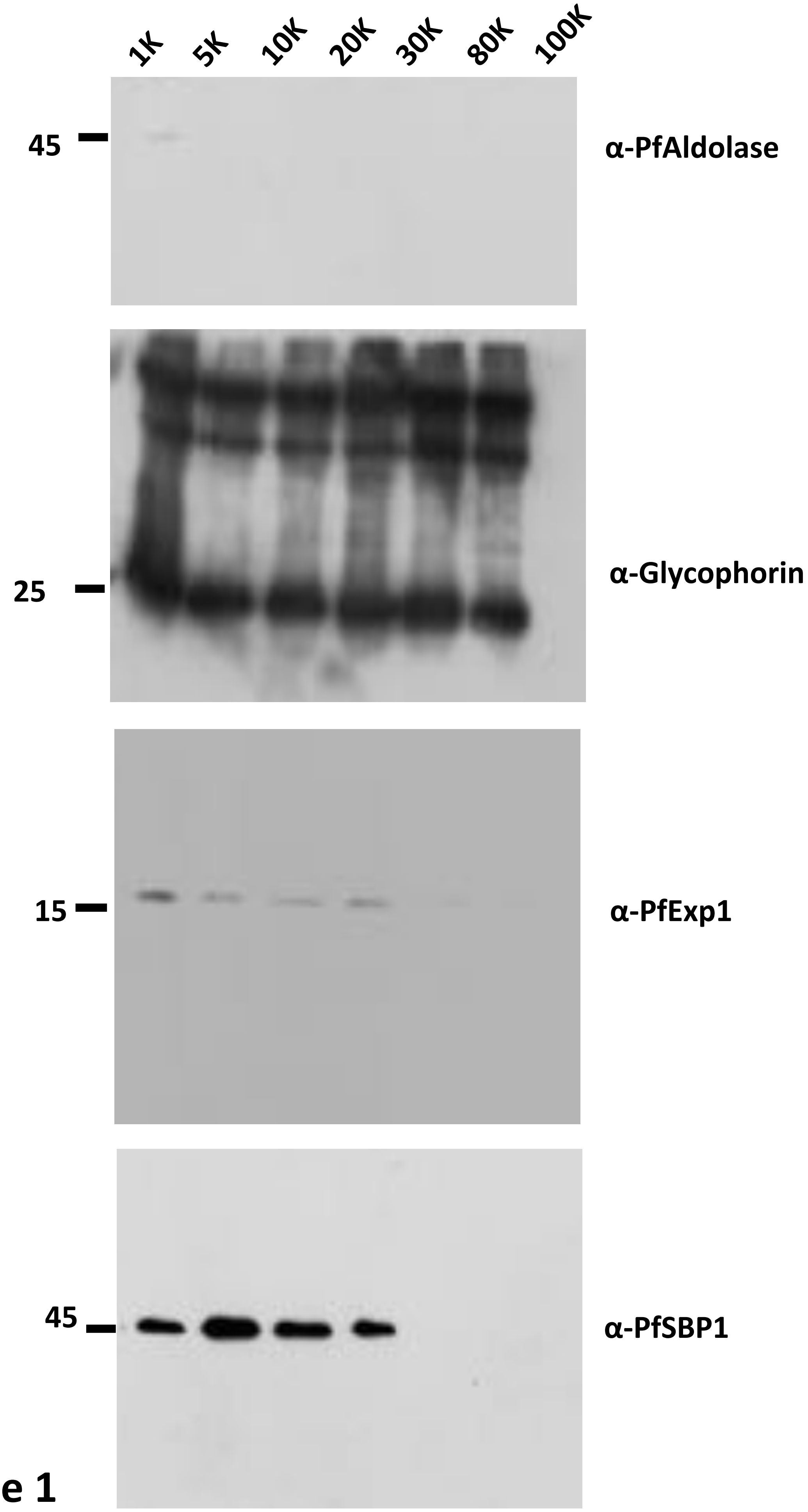

1K
5K
10K
20K
30K
80K
100K
45
α-PfAldolase
25
α-Glycophorin
15
α-PfExp1
45
α-PfSBP1

Figure 1

A

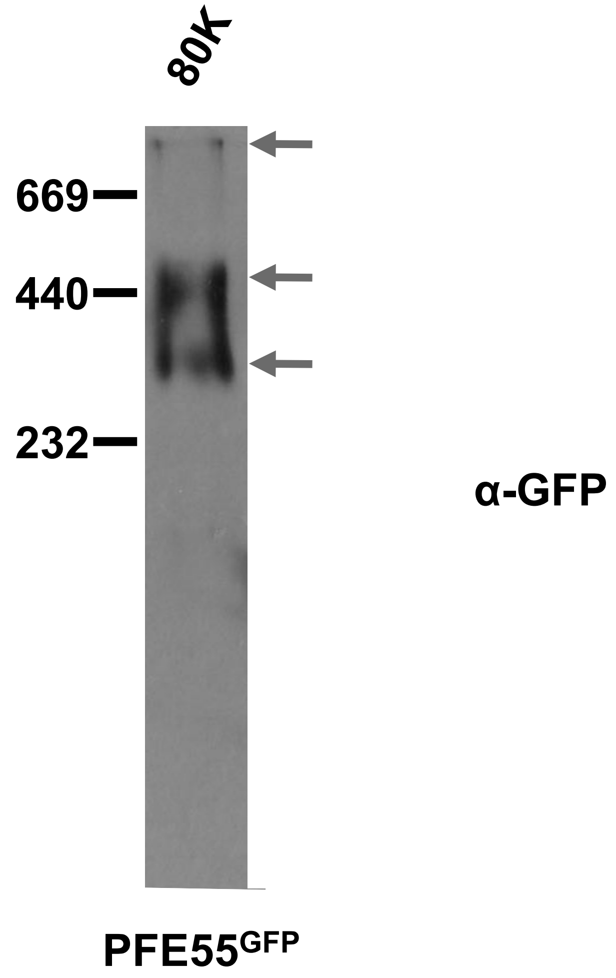

Figure 2

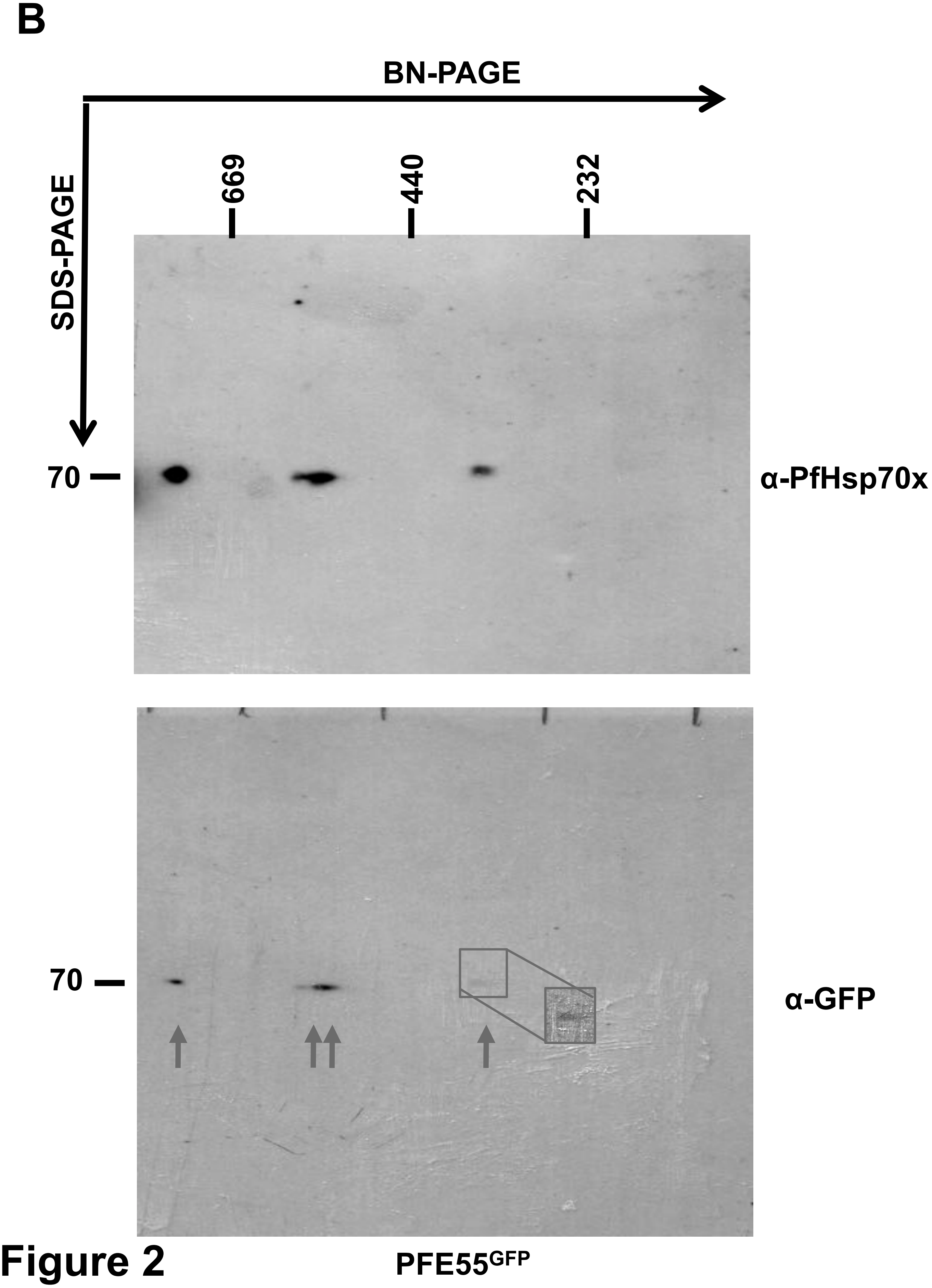

Figure 2

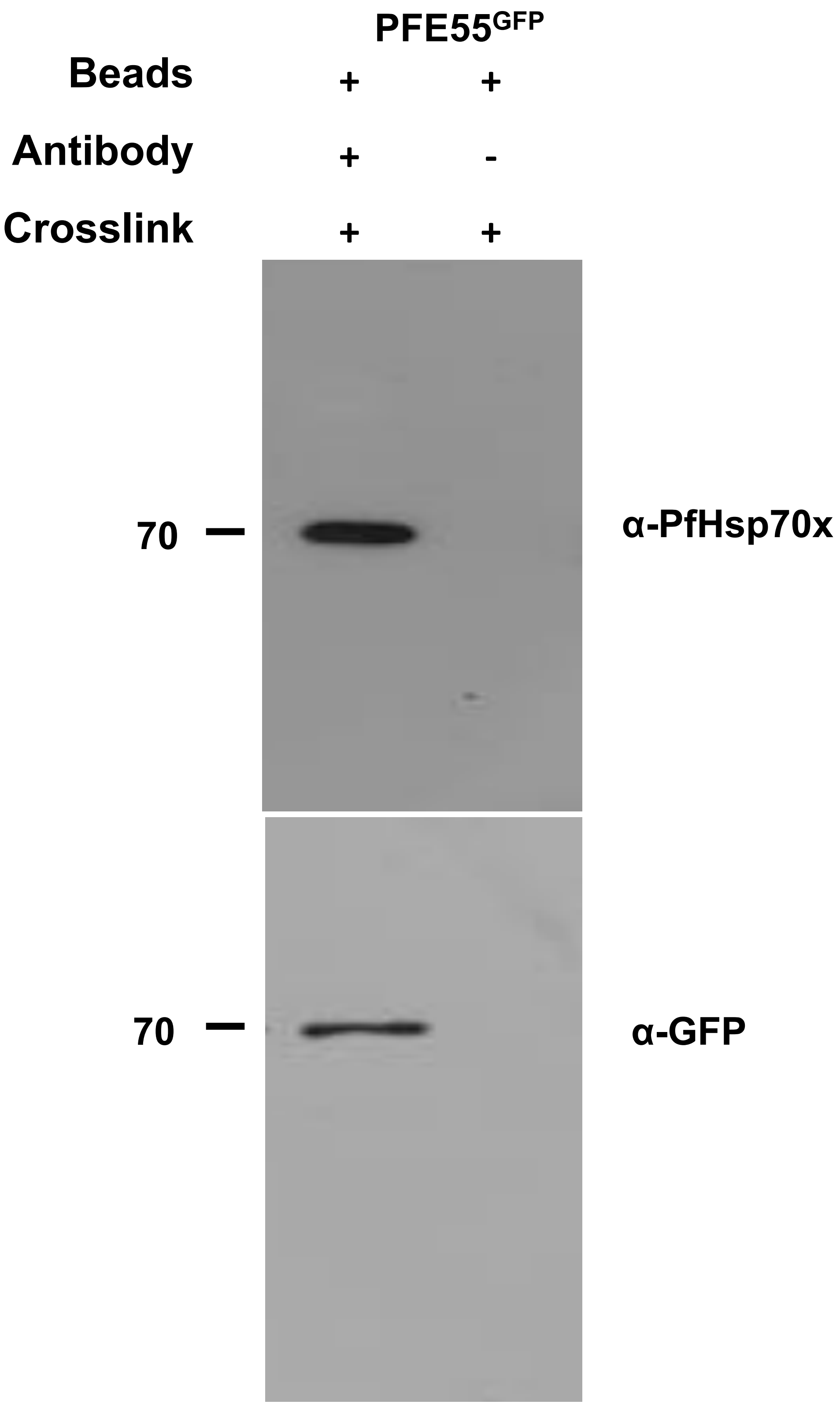

IP: α-PfHsp70x

Figure 3

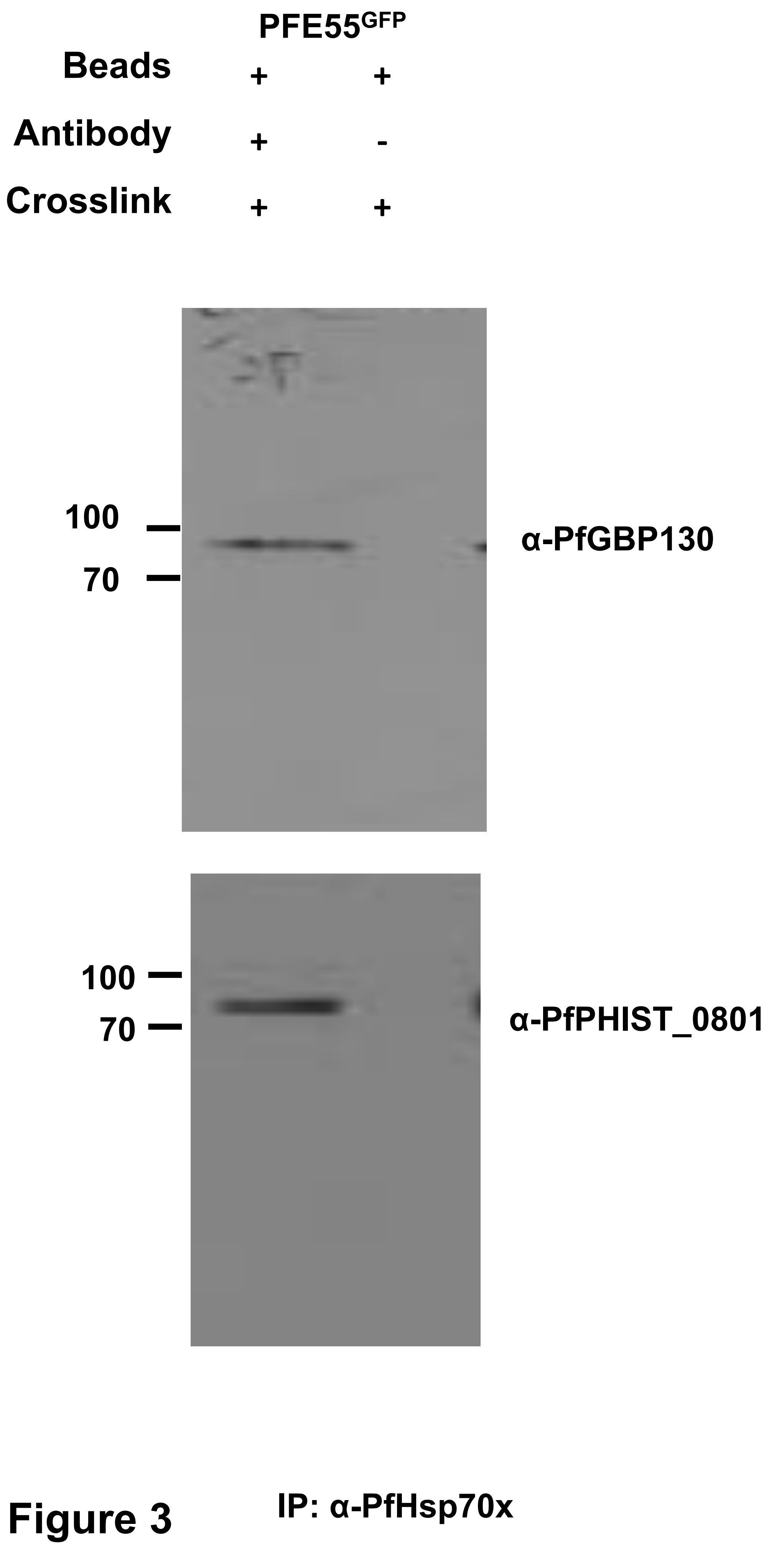
PFE55GFP
Beads + +
Antibody + -
Crosslink + +
100
70
α-PfGBP130
100
70
α-PfPHIST_0801
IP: α-PfHsp70x

Figure 3

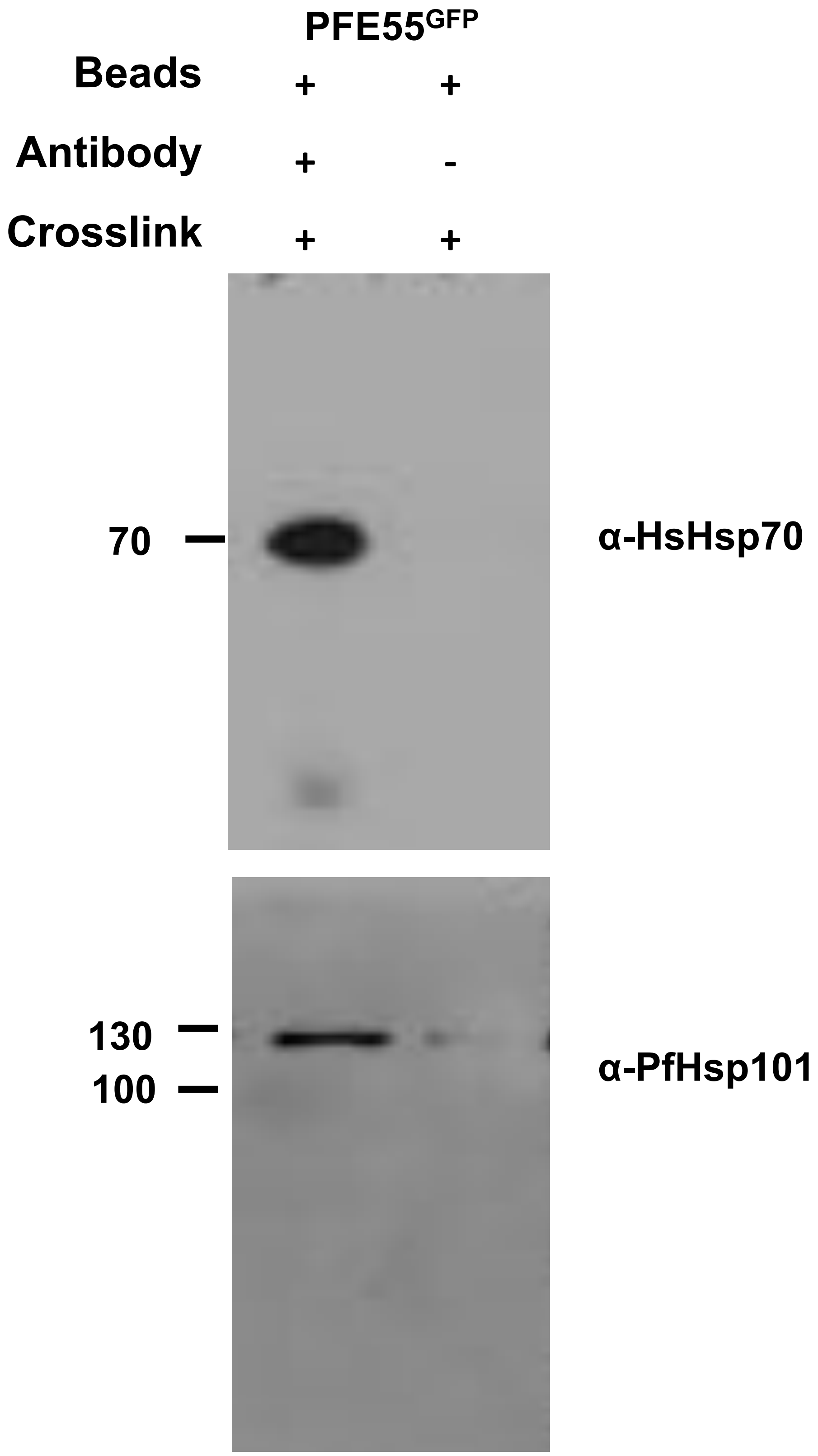

PFE55GFP
Beads + +
Antibody + -
Crosslink + +
70
α-HsHsp70
130
100
α-PfHsp101
IP: α-PfHsp70x

Figure 3

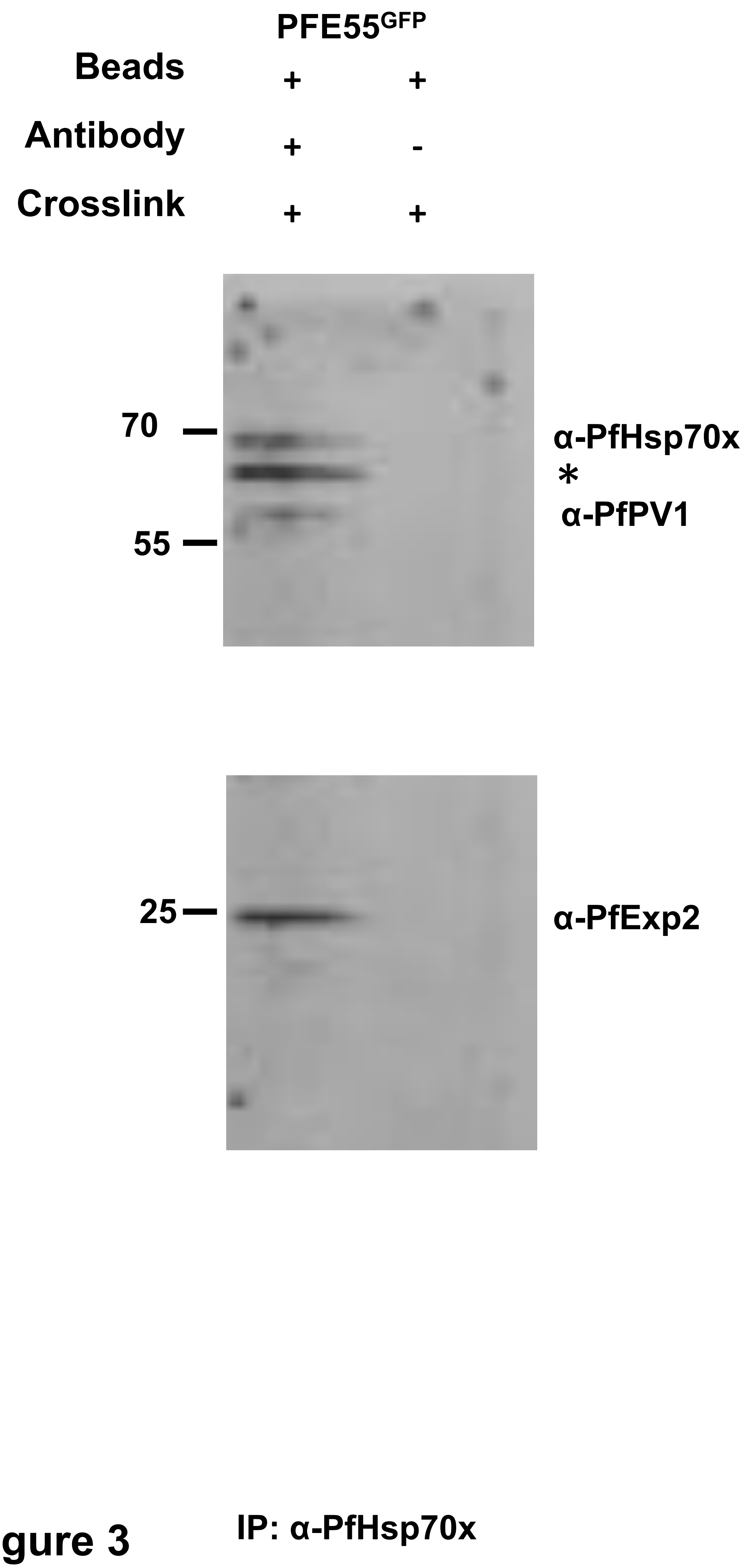
PFE55GFP
Beads + +
Antibody + -
Crosslink + +
70
55
α-PfHsp70x
*
α-PfPV1
25
α-PfExp2
IP: α-PfHsp70x

Figure 3

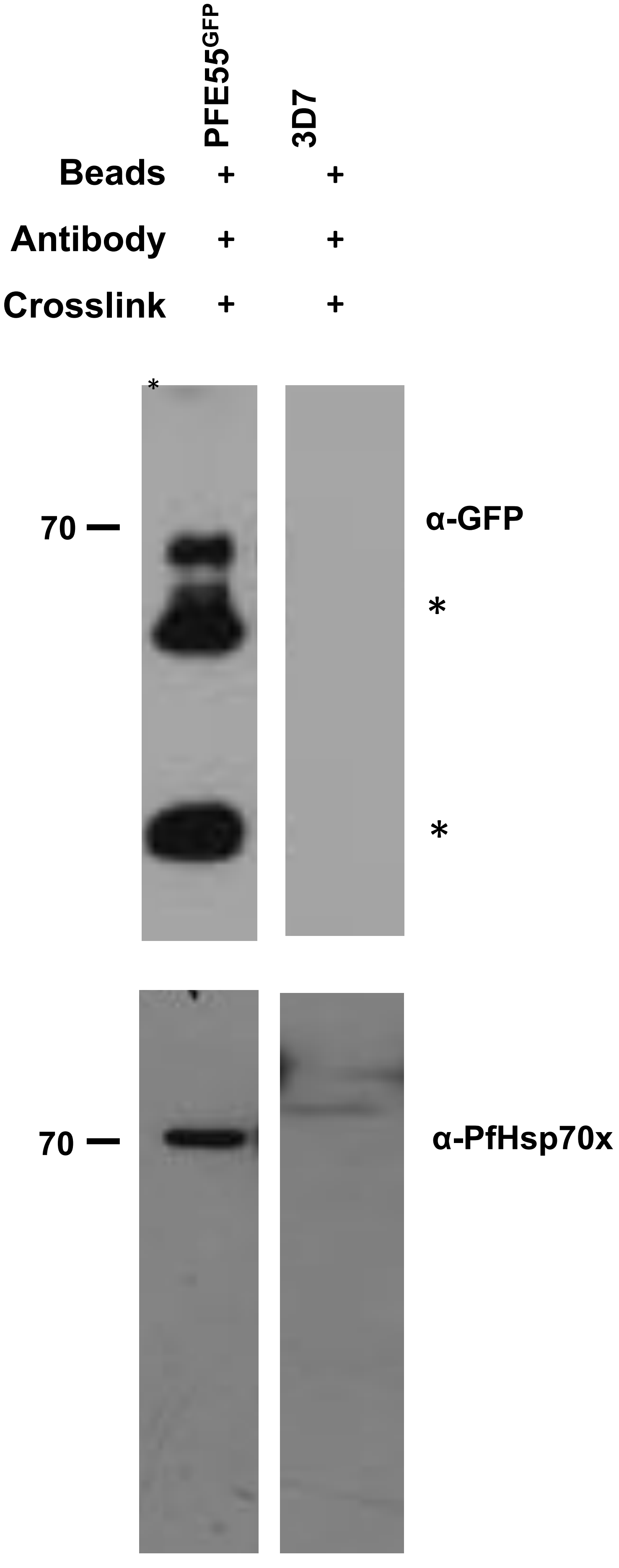

Figure 4 IP: α-GFP

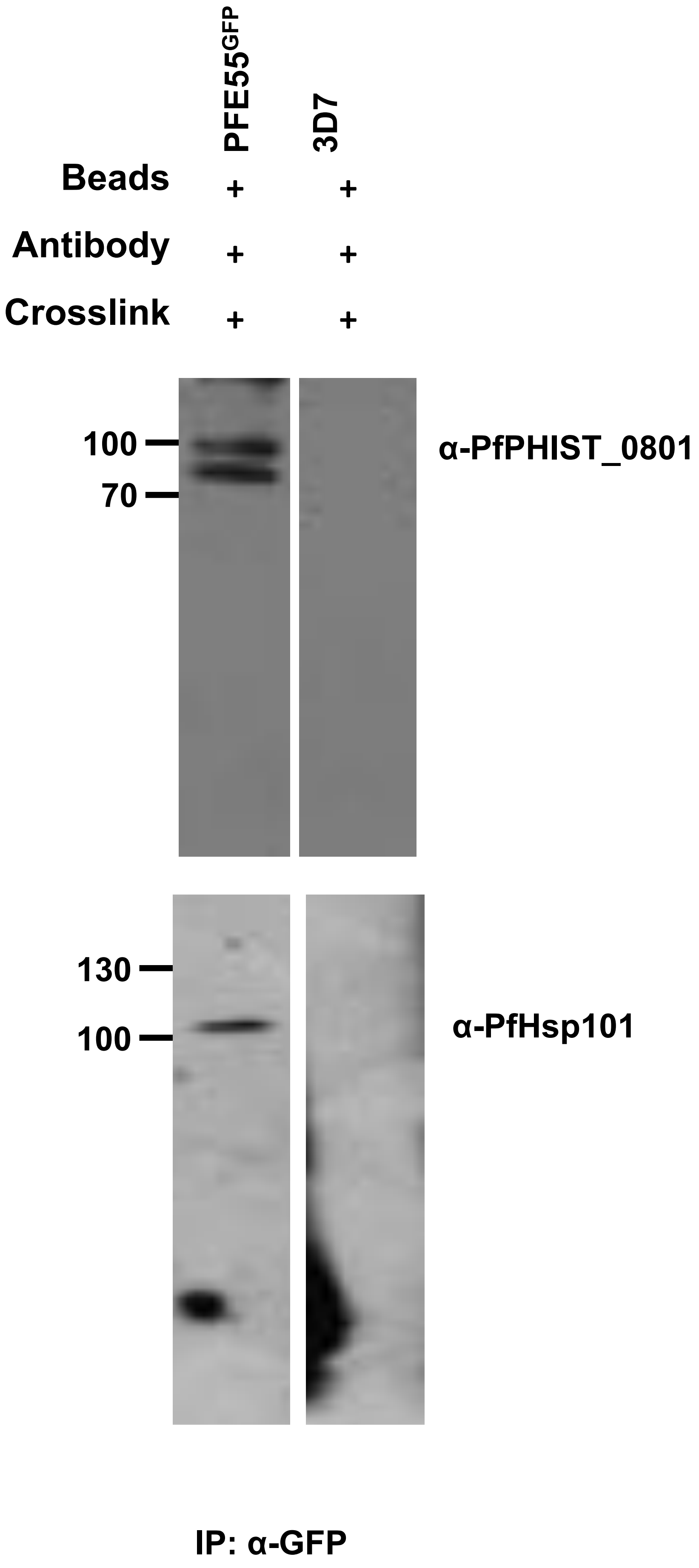

PFE55GFP
3D7
Beads + +
Antibody + +
Crosslink + +
100
70
α-PfPHIST_0801
130
100
α-PfHsp101
IP: α-GFP

Figure 4

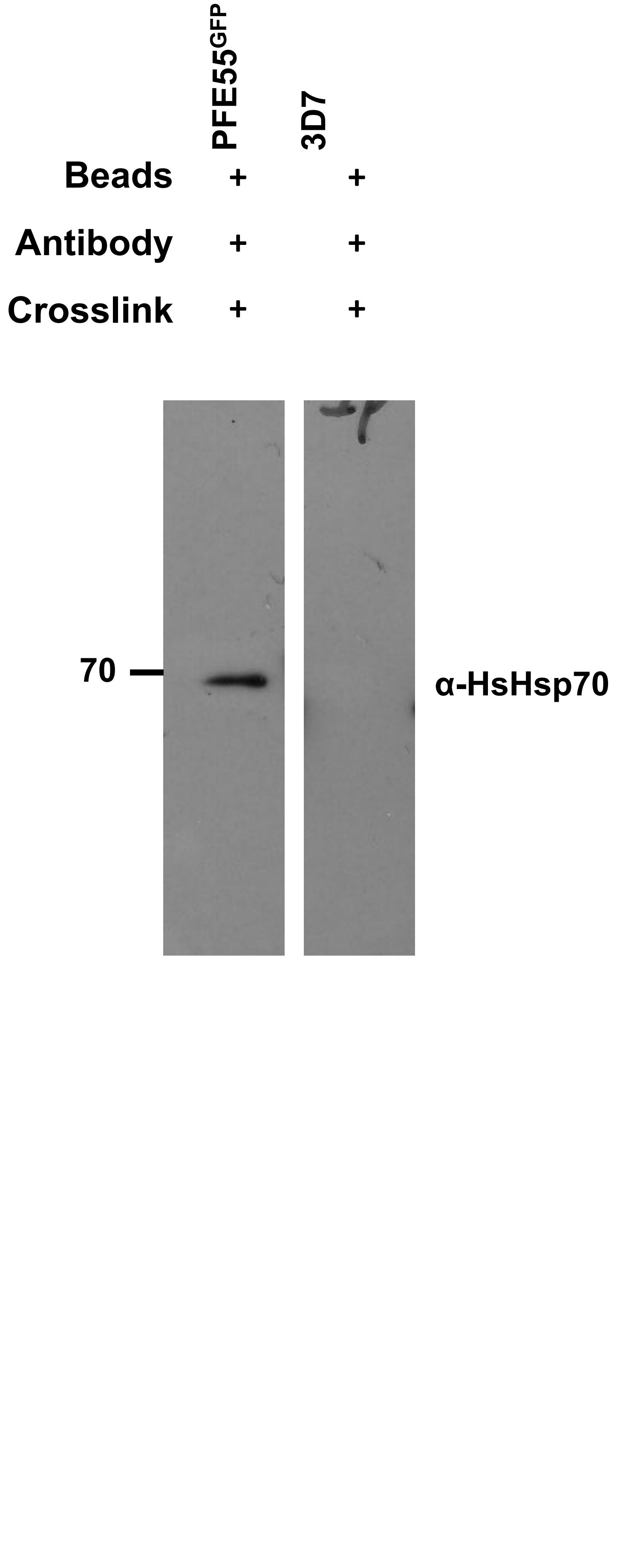

Figure 4

A

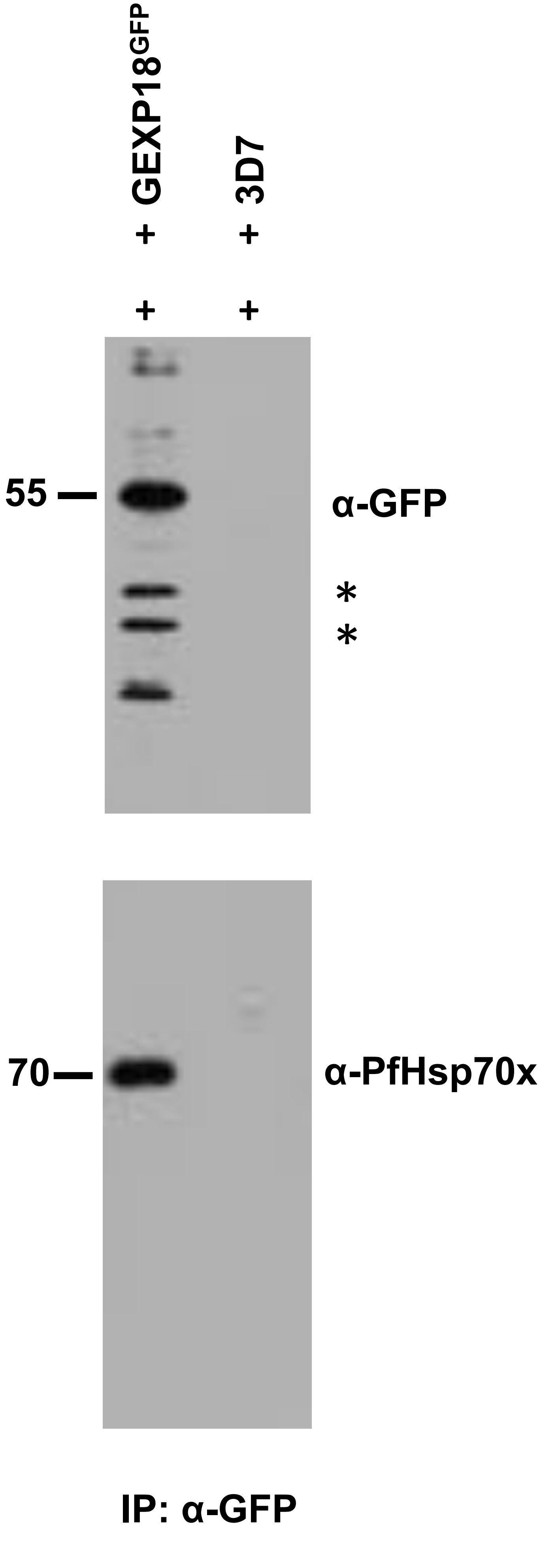

Figure 6

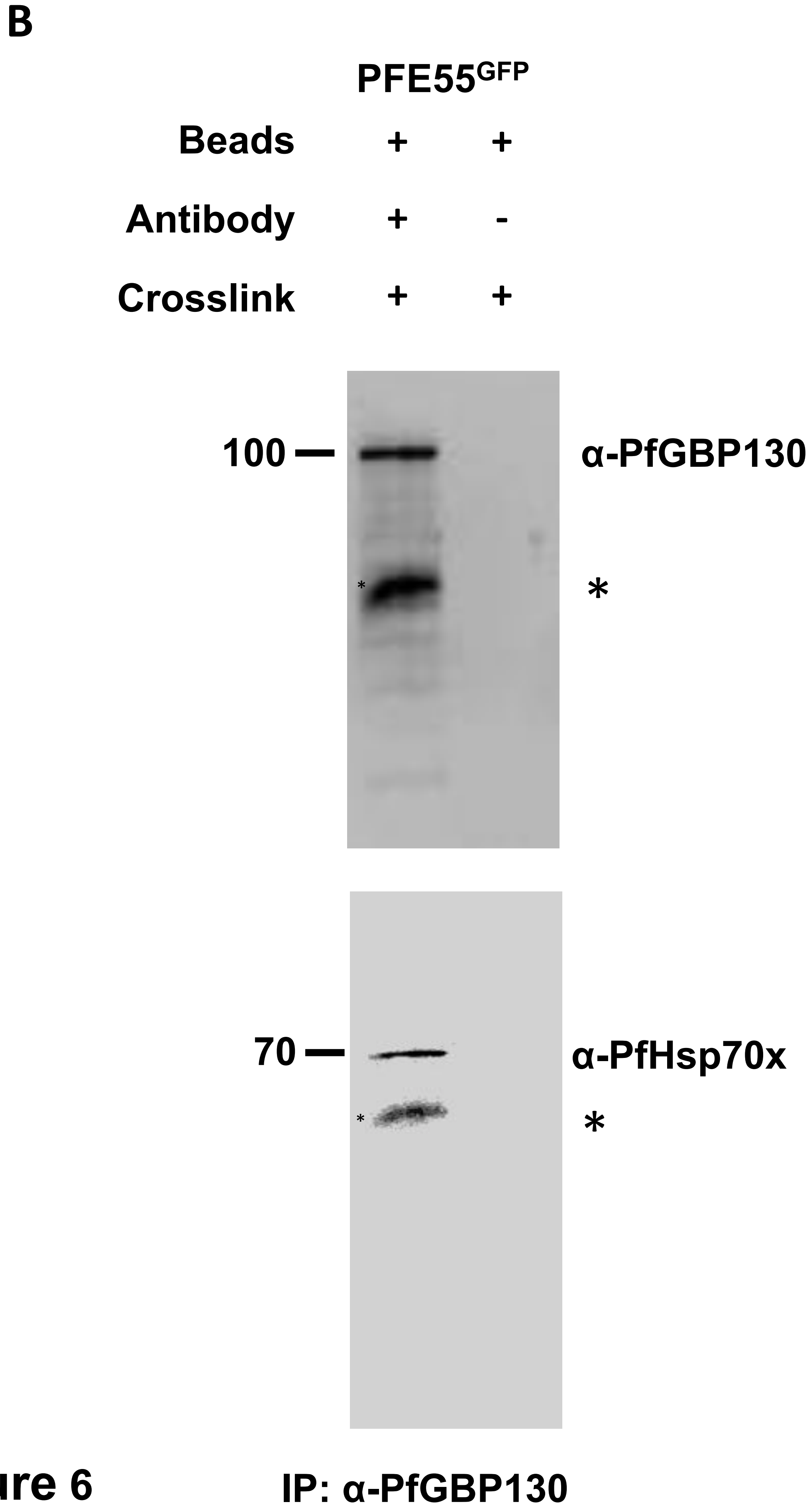
B
PFE55GFP
Beads + +
Antibody + -
Crosslink + +
100
α-PfGBP130
*
70
α-PfHsp70x
*
IP: α-PfGBP130

Figure 6

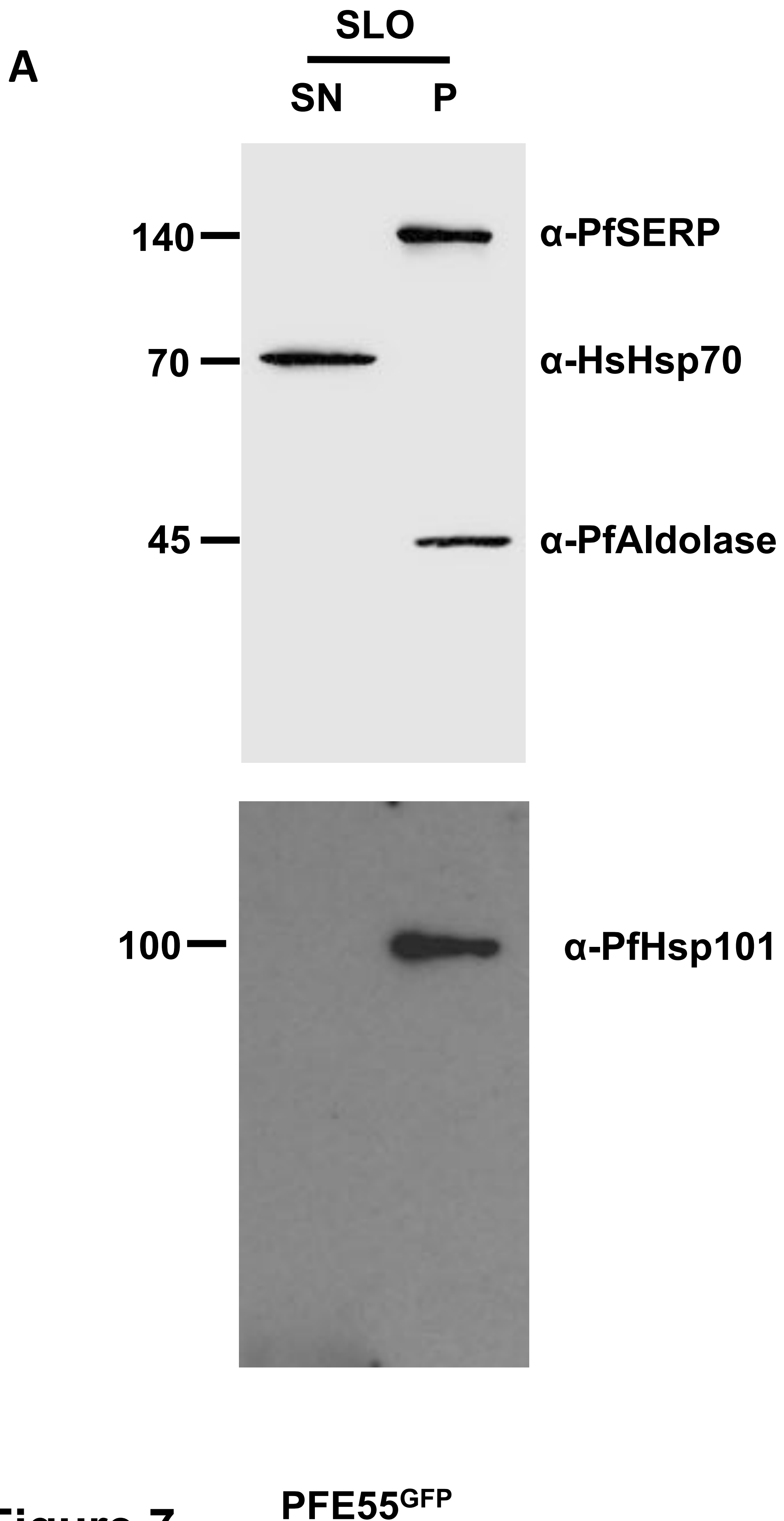
A
SLO
SN
P
140
70
45
α-PfSERP
α-HsHsp70
α-PfAldolase
100
α-PfHsp101
PFE55GFP

Figure 7

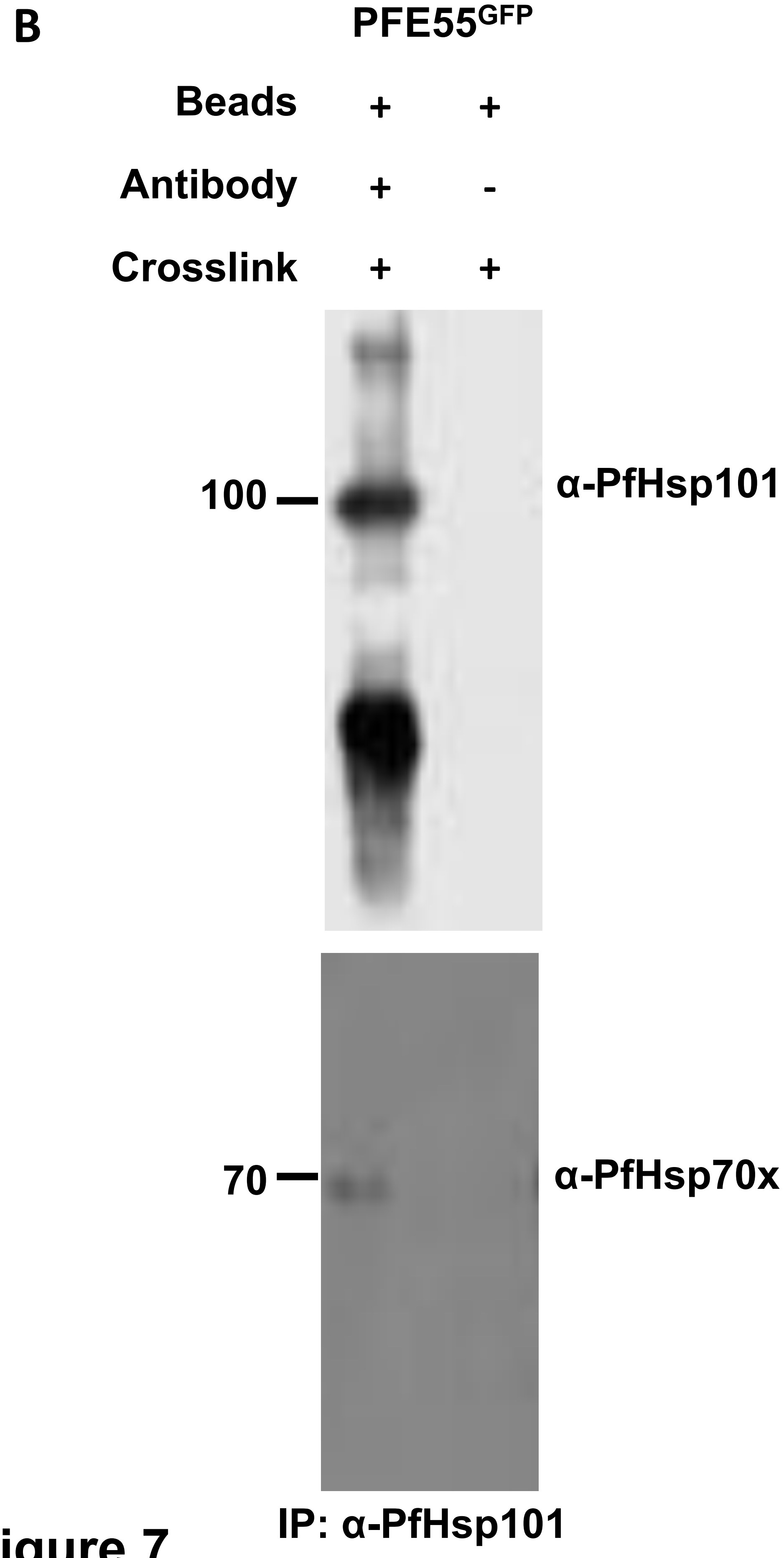
B
PFE55GFP
Beads + +
Antibody + -
Crosslink + +
100
α-PfHsp101
70
α-PfHsp70x
IP: α-PfHsp101

Figure 7

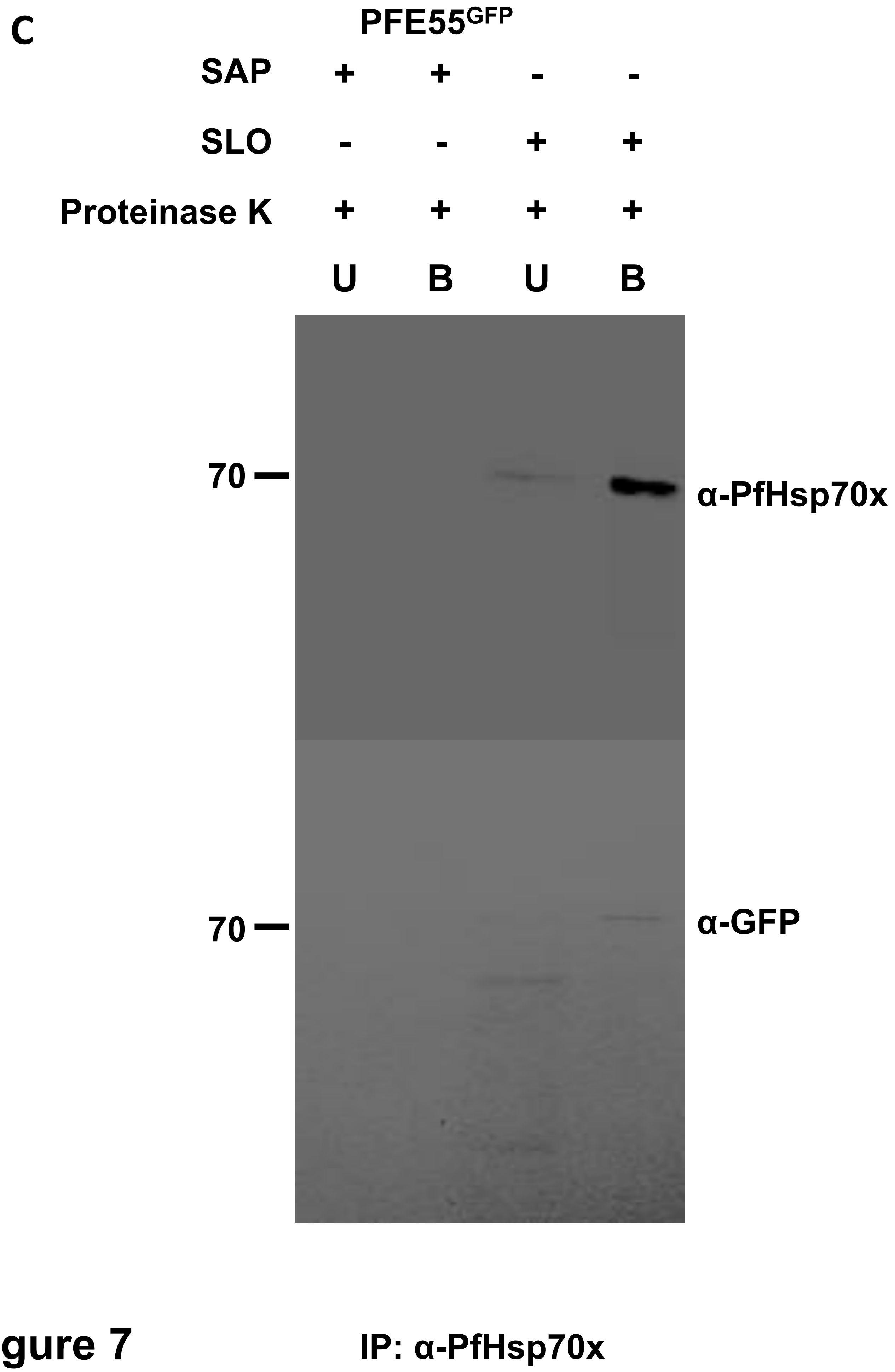
C
PFE55^{GFP}
SAP + + - -
SLO - - + +
Proteinase K + + + +
U B U B
70
α-PfHsp70x
70
α-GFP
IP: α-PfHsp70x

Figure 7

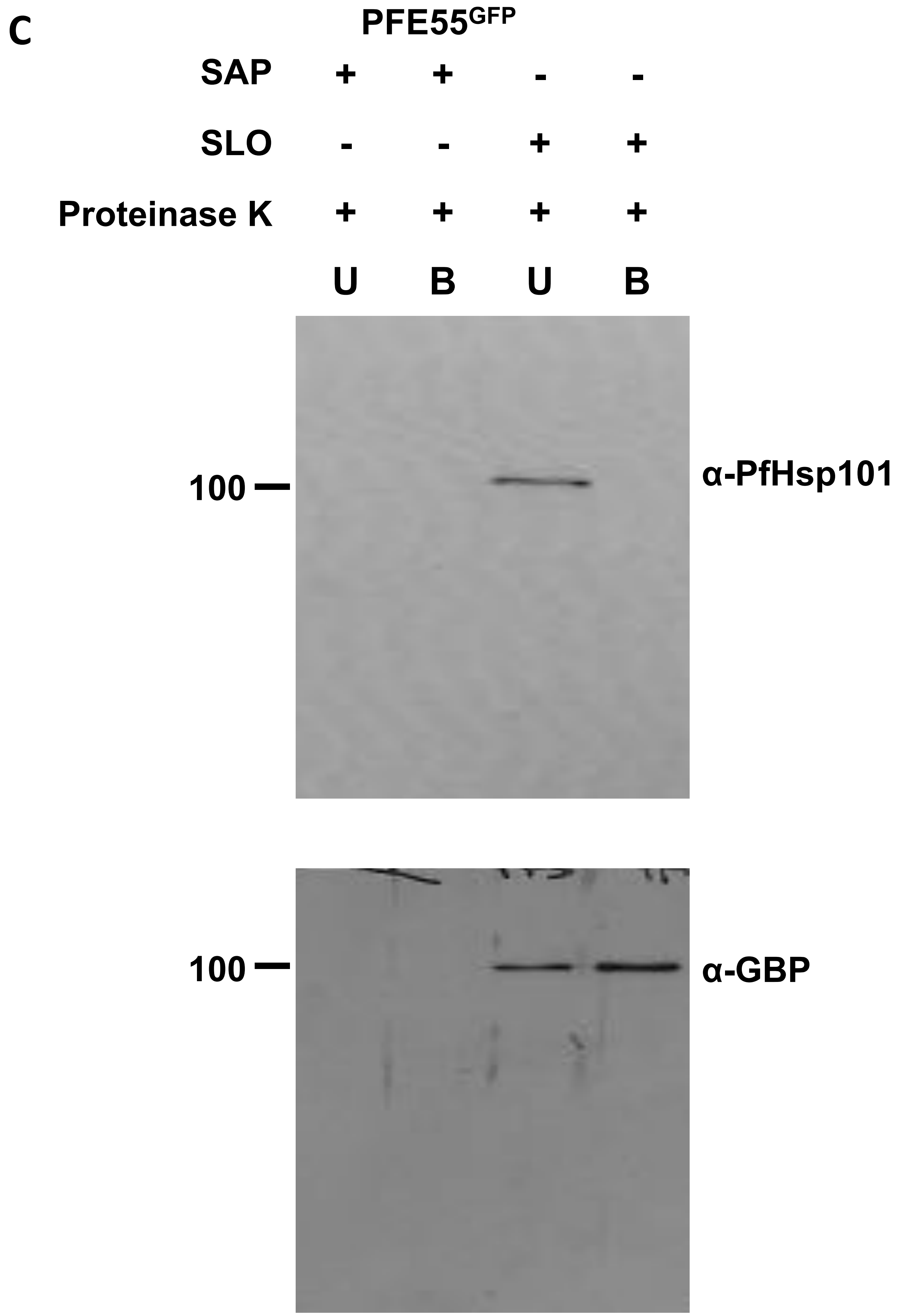

Figure 7

IP: α-PfHsp70x
